# Supplementary material for: Factors associated with use of long-acting reversible and permanent contraceptives among married women in rural Kenya: A community-based cross-sectional study in Kisii and Kilifi counties
Source: PLoS One. 2022 Oct 6;17(10):e0275575. doi: 10.1371/journal.pone.0275575 (PMC9536593; doi:10.1371/journal.pone.0275575)
Supplement: S1 File — (DOCX) [file pone.0275575.s001.docx]

## **Household Survey** **Questionnaire**

| **SECTION 1: HOUSEHOLD QUESTIONNAIRE - KENYA** | |
| --- | --- |
| household information panel HH | |
| **HH1**. Cluster number: ___ ___ ___ | **HH2**. Household number: ___ ___ |
| **HH3**. Interviewer’s name and number: |  |
| Name _________________________ ___ ___ |  |
| **HH5**. Day / Month / Year of interview:  ___ ___ /___ ___ / 2 0 1 ___ | **HH5A.** County:  Kilifi 01  Kisii 02 |
| **HH5D**. Is the household selected for Questionnaire for Men? | Yes 1  No 2 |
| READ THE CONSENT FORM (HARD COPY) TO THE RESPONDENT; TAKE CONSENT. ENSURE THAT THEY SIGN THE CONSENT FORM IF THEY AGREE TO PARTICIPATE”. | |
| **🞎** Yes, permission is given ⇨ *B*egin the interview.  **🞎** No, permission is not given ⇨ Circle 04 in HH6. Discuss this result with your supervisor. | |
| **HH6**. Result of household interview: Completed 01  No household member or no competent respondent at home at time of visit 02  Entire household absent for extended period of time 03  Refused 04  Dwelling vacant / Address not a dwelling 05  Dwelling destroyed 06  Dwelling not found 07  Other (specify) 96 | |

| *After the household questionnaire has been completed, fill in the following information:* |  |  |
| --- | --- | --- |
| **HH6A**. Respondent to Household Questionnaire:  Line number ___ ___ |  |  |
| **HH6B**. Total number of  household members: ___ ___ |  | *After all questionnaires for the household have been completed, fill in the following information:* |
| **HH6C**. Number of women  age 15-49 years: ___ ___ |  | **HH6D**. Number of women’s  questionnaires completed: ___ ___ |
| *If the household is selected for Questionnaire for Men:*  **HH6E**. Number of men  age 15-49 years: ___ ___ |  | *If the household is selected for Questionnaire for Men:*  **HH6F**. Number of men’s  questionnaires completed: ___ ___ |
| **HH6G**. Number of children  under age 5: ___ ___ |  | **HH6H**. Number of under-5  questionnaires completed: ___ ___ |

| list of household members HL |
| --- |
| First, please tell me the name of each person who usually lives here, starting with the head of the household.  List the head of the household in line 01. List all household members (HL2), their relationship to the household head (HL3), and their sex (HL4)  Then ask: Are there any others who live here, even if they are not at home now?  If yes, complete listing for questions HL2-HL4. Then, ask questions starting with HL5 for each person at a time.  Use an additional questionnaire if all rows in the List of Household Members have been used. |

b

| **HL1**.  Line  no. | **HL2**.  Name | **HL3**.  What is the relation-ship of (*name*) to the head of house-hold?  See below | **HL4**.  Is (*name*) male or female?  1 Male  2 Female | | **HL5**.  What is (*name*)’s date of birth? | | **HL6**.  How old is (*name*)?  *Record in completed years.*  *If age is 95 or above, record ‘95’.* | **HL6A**  Does (*name*) usually live here? | **HL6B**  Did (*name*) stay here last night? | **HL6C**  What is (*name*)’s marital status?  1-married  2-widowed  3-divorced/ separated  4-never married | **HL7A**.  For  Women -  age 15-49  Circle line no. if woman age 15-49 | **HL7B**.  For  Children  age 0-59 months  Circle line no. if age  0-59 months. | **HL8**.  For  Children  age 0-59 months  Is (*name*)’s natural mother alive?  1 Yes  2 No  8 DK | **HL9**.  Does (*name*)’s natural mother live in this house-hold?  If “Yes”,  record  line no.  of  mother.  If “No”, record 00. | **HL10.**  For Children age 0-59 months  Record Line no. of mother from HL9 if indicated. If HL9 is blank or “00” ask: Who is the primary caretaker of (Name) | **HL9A.**  Does (*name*)’s natural father live in this house-hold?  If “Yes”, record line no. of FATHER.  If “No”, record 00. | **HL10A.**  For  Men age 15-54  Circle line no. if man age 15-54 who are  *married, widowed or divorced or separated*  and the house-hold is selected for Questionnaire for Men. |
| --- | --- | --- | --- | --- | --- | --- | --- | --- | --- | --- | --- | --- | --- | --- | --- | --- | --- |
|  |  |  |  |  | 98 DK | 9998 DK |  |  |  |  |  |  |  |  |  |  |  |
| Line | Name | Relation* | M | F | Month | Year | Age | Y N | Y N |  | 15-49 | 0-4 | y n dk | Mother | Mother | Father | Father |
| 01 |  | **0 1** | 1 | 2 | __ __ | __ __ __ __ | __ __ | 1 2 | 1 2 |  | 01 | 01 | ___ ___ |  |  |  | 01 |
| 02 |  | ___ ___ | 1 | 2 | __ __ | __ __ __ __ | __ __ | 1 2 | 1 2 |  | 02 | 02 | ___ ___ |  |  |  | 02 |
| 03 |  | ___ ___ | 1 | 2 | __ __ | __ __ __ __ | __ __ | 1 2 | 1 2 |  | 03 | 03 | ___ ___ |  |  |  | 03 |
| 04 |  | ___ ___ | 1 | 2 | __ __ | __ __ __ __ | __ __ | 1 2 | 1 2 |  | 04 | 04 | ___ ___ |  |  |  | 04 |
| 05 |  | ___ ___ | 1 | 2 | __ __ | __ __ __ __ | __ __ | 1 2 | 1 2 |  | 05 | 05 | ___ ___ |  |  |  | 05 |
| 06 |  | ___ ___ | 1 | 2 | __ __ | __ __ __ __ | __ __ | 1 2 | 1 2 |  | 06 | 06 | ___ ___ |  |  |  | 06 |
| 07 |  | ___ ___ | 1 | 2 | __ __ | __ __ __ __ | __ __ | 1 2 | 1 2 |  | 07 | 07 | ___ ___ |  |  |  | 07 |
| 08 |  | ___ ___ | 1 | 2 | __ __ | __ __ __ __ | __ __ | 1 2 | 1 2 |  | 08 | 08 | ___ ___ |  |  |  | 08 |
| 09 |  | ___ ___ | 1 | 2 | __ __ | __ __ __ __ | __ __ | 1 2 | 1 2 |  | 09 | 09 | ___ ___ |  |  |  | 09 |
| 10 |  | ___ ___ | 1 | 2 | __ __ | __ __ __ __ | __ __ | 1 2 | 1 2 |  | 10 | 10 | ___ ___ |  |  |  | 10 |
| 11 |  | ___ ___ | 1 | 2 | __ __ | __ __ __ __ | __ __ | 1 2 | 1 2 |  | 11 | 11 | ___ ___ |  |  |  | 11 |
| 12 |  | ___ ___ | 1 | 2 | __ __ | __ __ __ __ | __ __ | 1 2 | 1 2 |  | 12 | 12 | ___ ___ |  |  |  | 12 |
| 13 |  | ___ ___ | 1 | 2 | __ __ | __ __ __ __ | __ __ | 1 2 | 1 2 |  | 13 | 13 | ___ ___ |  |  |  | 13 |
| 14 |  | ___ ___ | 1 | 2 | __ __ | __ __ __ __ | __ __ | 1 2 | 1 2 |  | 14 | 14 | ___ ___ |  |  |  | 14 |

| Probe for additional household members.  Probe especially for any infants or small children not listed, and others who may not be members of the family (such as servants, friends) but who usually live in the household.  Insert names of additional members in the household list and complete form accordingly. |
| --- |

| Now for each woman age 15-49 years, write her name and line number and other identifying information in the information panel of a separate Individual Women’s Questionnaire.  For each man age 15-49 years, write his name and line number and other identifying information in the information panel of a separate Individual Man’s Questionnaire.  For each child under age 5, write his/her name and line number AND the line number of his/her mother or caretaker in the information panel of a separate Under-5 Questionnaire.  You should now have a separate questionnaire for each eligible woman, each eligible man, and each child under five in the household. |
| --- |

| * *Codes for* ***HL3****: Relationship to head of household:* | 01 Head  02 Spouse / Partner  03 Son / Daughter | 04 Son-In-Law / Daughter-In-Law  05 Grandchild  06 Parent | 07 Parent-In-Law  08 Brother / Sister  09 Brother-In-Law / Sister-In-Law | 10 Uncle / Aunt  11 Niece / Nephew  12 Other relative | 13 Adopted / Foster/ Stepchild  14 Servant (Live-in) | 96 Other (Not related)  98 DK |
| --- | --- | --- | --- | --- | --- | --- |

| **HOUSEHOLD ASSETS MODULE (HA)** | | |
| --- | --- | --- |
| 1. What type of fuel does your household mainly use for cooking? | Electricity 01  Liquefied Petroleum Gas (LPG) 02  Natural gas 03  Biogas 04  Paraffin/Kerosene 05  Charcoal 06  Coal / Lignite 07  Wood 08  Straw / Shrubs / Grass 09  Agricultural crop residue 10  Animal dung 11  No food cooked in household 95  Other (specify)­­­­­­­_____________________96 |  |
| 1. How many rooms in this household can be used for sleeping? | Rooms ___ ___ |  |
| 1. Does this household own any livestock, herds, other farm animals, or poultry? | Yes 1  No 2  Don’t Know____________________________98 | 2🡪HA5  98🡪HA5 |
| 1. How many of the following animals does this household own?   READ EACH ITEM. IF NONE, RECORD 00.  IF 95 OR MORE, RECORD 95.  Local cattle (indigenous)?  Exotic/grade cattle? Horses/donkeys/camels?  Goats?  Sheep?  Chickens? | Local cattle  Exotic/grade cattle ___ ___  Horses/donkeys/camels ___ ___  Goats ___ ___  Sheep ___ ___  Chickens ___ ___ |  |
| 1. Does any member of this household own agricultural land? | Yes 1  No ----------------------------------------------------- 2  Don’t know ----------------------------------- 9 | 2🡪HA7  98🡪HA7 |
| 1. How many acres of agricultural land do members of this household own? | Acres **__ __. __** |  |
| 1. Does your household have: 2. Electricity? 3. A radio? 4. A television? 5. A refrigerator/Freezer? 6. Solar panel 7. table? 8. chair? 9. Bed? 10. Cupboard? 11. Clock 12. DVD player? 13. Cassette or CD Player? | Yes No  Electricity 1 2  Radio 1 2  Television 1 2  Refrigerator/Freezer 1 2  Solar Panel 1 2  Table 1 2  Chair 1 2  Bed 1 2  Cupboard 1 2  Clock 1 2  DVD Player 1 2  Cassette or CD Player 1 2 |  |
| **HA7B. Does your household have any of the following? (to be asked to Kisii county only)**  [A] Posho-Mill  [B] Sugar Cane Crusher  [C] Power Saw | Yes No  Posho-Mill 1 2  Sugar Cane Crusher 1 2  Power Saw 1 2 |  |
| 1. Does any member of your household own:   [A] A watch?  [B] A bicycle?  [C] A mobile telephone?  [D] A motorcycle or scooter?  [E] A car or truck? | Yes No  Watch 1 2  Bicycle 1 2  Mobile telephone 1 2  Motorcycle / Scooter 1 2  Car / Truck 1 2 |  |
| **HA9A.** Main material of the dwelling floor.  Record observation. | Natural floor  Earth / Sand 11  Dung 12  Rudimentary floor  Wood planks 21  Palm / Bamboo 22  Finished floor  Parquet or polished wood 31  Vinyl or asphalt strips 32  Ceramic tiles 33  Cement 34  Carpet 35  Other (specify) 96 |  |
| **HA9B.** Main material of the roof.  Record observation. | Natural roofing  No Roof 11  Thatch / Grass / Makuti 12  Dung / Mud / Sod 13  Rudimentary roofing  Iron Sheets 21  Tin cans 22  Finished roofing  Asbestos Sheet 31  Concrete 32  Tiles 33  Other (specify) 96 |  |
| **HA9C.** Main material of the external walls.  Record observation. | Natural walls  No walls 11  Cane / Palm / Trunks 12  Dung / Mud / Sod 13  Rudimentary walls  Bamboo with mud 21  Stone with mud 22  Uncovered adobe 23  Plywood 24  Cardboard 25  Reused wood 26  Iron Sheets 27  Finished walls  Cement 31  Stone with lime / cement 32  Bricks 33  Cement blocks 34  Covered adobe 35  Wood planks / shingles 36  Other (specify) 96 |  |

| **VECTOR CONTROL MODULE (VC)** | | |
| --- | --- | --- |
| 1. At any time in the past 12 months, has anyone come into your dwelling to spray the interior walls against mosquitoes? | Yes 1  No 2  Don’t know 98 | 2🡪VC3  98🡪VC3 |
| 1. Who sprayed the dwelling? | GOVERNMENT WORKER/PROGRAM 1  PRIVATE COMPANY 2  NONGOVERNMENTAL ORGANIZATION (NGO) 3  Other (specify) 4  Don’t know 98 |  |
| 1. Does your household have any mosquito nets that can be used while sleeping? | Yes 1  No 2  Don’t know 98 | 2🡪Next module  98🡪Next module |
| 1. How many mosquito nets does your household have?   **Ask** ‘Can you show the nets to me, please?’ | Number of nets: _____ |  |

| **Ask the following questions for each net. If more than 3 nets, use additional questionnaires.**  **Check here if additional questionnaire(s) used: 🞎** | | | | |
| --- | --- | --- | --- | --- |
|  | **Net 1** | **Net 2** | **Net 3** |  |
| **VC5.** | Observed 1  Not observed 2 | Observed 1  Not observed 2 | Observed 1  Not observed 2 |  |
| 1. How many months ago was this net obtained?   **If less than 1 month, record ‘00’** | __ __ Months ago  More than 36 months ago 95  Don’t know 98 | __ __ Months ago  More than 36 months ago 95  Don’t know 98 | __ __ Months ago  More than 36 months ago 95  Don’t know 98 |  |
| 1. What brand is this net?   **Observe the net or ask for the type/brand of the net. If the respondent does not know the type/brand and the net cannot be observed, show pictures** | Long-lasting insecticide-treated net  PERMANET 11  OLISET 12  DURANET 13  NETPROTECT 14  INTERCEPTOR 15  Other (*specify*) 16  Insecticide-treated net  PERMETHRINE 21  DELTA METHRINE 22  CYFULTRINE 23  Other (*specify*) 26  Not pre-treated (*specify brand*) 31  Don’t know/not sure 98 | Long-lasting insecticide-treated net  PERMANET 11  OLISET 12  DURANET 13  NETPROTECT 14  INTERCEPTOR 15  Other (*specify*) 16  Insecticide-treated net  PERMETHRINE 21  DELTA METHRINE 22  CYFULTRINE 23  Other (*specify*) 26  Not pre-treated (*specify brand*) 31  Don’t know/not sure 98 | Long-lasting insecticide-treated net  PERMANET 11  OLISET 12  DURANET 13  NETPROTECT 14  INTERCEPTOR 15  Other (*specify*) 16  Insecticide-treated net  PERMETHRINE 21  DELTA METHRINE 22  CYFULTRINE 23  Other (*specify*) 26  Not pre-treated (*specify brand*) 31  Don’t know/not sure 98 |  |
| 1. When you got this net, was it already treated with an insecticide to kill or repel mosquitoes? | Yes 1  No 2  Don’t know 98 | Yes 1  No 2  Don’t know 98 | Yes 1  No 2  Don’t know 98 |  |
| 1. Did anyone sleep under this net last night? | Yes 1  No 2**🡪Next module**  Don’t know 98**🡪 Next module** | Yes 1  No 2**🡪 Next module**  Don’t know 98**🡪 Next module** | Yes 1  No 2**🡪 Next module**  Don’t know 98**🡪 Next module** |  |
| 1. Who slept under this net last night?   **Record the name(s) and line number(s) of the person(s) from the household listing** | Name  Line no.: ___ ___  Name  Line no.: ___ ___  Name  Line no.: ___ ___  Name  Line no.: ___ ___ | Name  Line no.: ___ ___  Name  Line no.: ___ ___  Name  Line no.: ___ ___  Name  Line no.: ___ ___ | Name  Line no.: ___ ___  Name  Line no.: ___ ___  Name  Line no.: ___ ___  Name  Line no.: ___ ___ |  |

| \| **SECTION 2: WOMEN’S questionnaire**  **Women’s Information PANEL** \| \| \| --- \| --- \| \|  \| \| \|  \| \| \| *This questionnaire is to be administered to all women age 15 through 49 (see List of Household Members, column HL7). A separate questionnaire should be used for each eligible woman.* \| \| \| **WM1**. Cluster number: \| **WM2**. Household number: \| \| ___ ___ ___ \| ___ ___ \| \| **WM3**. Woman’s name: \| **WM4**. Woman’s line number: \| \| Name \| ___ ___ \| \| **WM5.** Check HL6 and record woman’s age: ___________________ \| \| \| **WM5A.** Interviewer’s name and number: \| **WM5B.** Day / Month / Year of interview: \| \| Name ___ ___ \| ___ ___ /___ ___ / 2 0 1 ___ \|  \| *Repeat consent process if not already read to this woman:*  READ THE CONSENT FORM (HARD COPY) TO THE RESPONDENT; TAKE CONSENT. ENSURE THAT THEY SIGN THE CONSENT FORM IF THEY AGREE TO participate AND LEAVE ASIGNED COPY OF CONSENT/ASSENT FORM WITH THE PARTICIPANTS”. \| \| --- \| \| *If greeting at the beginning of the household questionnaire has already been read to this woman, then read the following:*  Now I would like to talk to you more about your health and other topics. This interview will take about ***insert number*** minutes. Again, all the information we obtain will remain strictly confidential and anonymous. \| \| **If the woman is 15-17 years old and married, read the consent script to any person the woman is comfortable with and the assent script to the woman. You MUST obtain the consent of the parent or guardian and assent of the woman before proceeding**  **If the woman is married, or is unmarried and older than 17 years, read her the woman’s consent script. You MUST obtain the woman’s consent before proceeding**  May I start now?   - *Yes, permission is given* ⇨ *Begin the interview.* - *No, permission is not given* ⇨ *Circle “05” in WM6C. Discuss this result with your supervisor.* \|  \| **WM6C**. Result of woman’s interview \| \| Completed 01  Not at home 02  Not competent 03  Deferred 04  Refused 05  Partially completed 06  Other (*specify*) 96 \| \| \| \| --- \| --- \| --- \| --- \| --- \| \| WM8. Time at beginning of interview \| \| __ __ hr __ __ min \| \| \| \| WM9. Time at end of interview \| \| __ __ hr __ __ min \| \| \| \| **WM10. During this interview is privacy ensured?**  **Probe: Was anyone else present and listening during the interview other than the woman being interviewed?** \| Yes 1  No. 2 \| \| 1🡪skip WM11 \| \| **WM11.Who else was present during the interview?**  **select all that apply**  **Probe: Was anyone else present and listening during the interview other than the woman being interviewed?** \| Husband/Partner 1  Children. 2  Other male relatives 3  Other female relatives. 4  Other (specify) 96 \| \|  \| \|  \|  \| \| \| \| --- \| --- \| --- \| \| **WOMAN’S INFORMATION MODULE (WI)** \| \| \| \| 1. In what month and year were you born? \| Date of birth Month __ __  DK month 98  Year __ __ __ __  DK year 9998 \|  \| \| 1. How old are you?   *Probe:* How old were you at your last birthday?  *Compare and correct W1 and/or W2 if inconsistent.* \| Age (in completed years) __ __ \|  \| \| **WI2A.** What is the highest level of school you attended? \| Preschool 0  Primary 1  Secondary 2  Higher 3  Did not attend school 05 \| 0**🡪**WI2C \| \| **WI2B.** What is the highest grade you completed at that level?  *If the first grade at this level is not completed,  enter “00”.* \| Grade __ __ \|  \| \| **WI2C.** Are you currently married or living together with a man as if married? \| Yes, currently married 1  Yes, living with a man 2  No, not in union 3 \| if WI2C=3 skip to WI2F \| \| **WI2D.** Now I would like to ask about your (first) (husband/partner). In what month and year did you start living with him? \| Month ___ ___  Don’t know month 98  Year ___ ___ ___ ___  Don’t know year 9998 \|  \| \| **WI2E.** Can you please identify your partner from this listing?  *Review the household listing and write down the line number of the partner* \| Line number __ __  Husband/partner not living in household 98 \|  \| \| **WI2F.** Who usually makes decisions about making major household purchases?  Select one \| Respondent 1  Husband/Partner 2  Respondent and Husband/Partner 3  Father or Father-in-law 4  Mother or Mother in-law 5  Other male family member 6  Other female family member 7  Other (*specify*) 96 \|  \| \| **WI2G.** Who usually makes decisions about health care for yourself?  Select one \| Respondent 1  Husband/Partner 2  Respondent and Husband/Partner 3  Father or Father-in-law 4  Mother or Mother in-law 5  Other male family member 6  Other female family member 7  Other (*specify*) 96 \|  \| \| **WI2H.** Who usually makes decisions about health care for your children \| Respondent 1  Husband/Partner 2  Respondent and Husband/Partner 3  Father or Father-in-law 4  Mother or Mother in-law 5  Other male family member 6  Other female family member 7  Not applicable 65  Prefer not to answer 66  Other (*specify*) 96 \| Skip if woman doesn’t have any children \| \| **WI2I** NOW I WOULD LIKE TO ASK ABOUT your knowledge regarding age of marriage, REGARDLESS if you are CURRENTLY married or not or AT WHAT AGE YOU WERE MARRIED.  what are issues or CONSEQUENCES OF early marriage that you have heard of?  **DO NOT PROMPT.**  **CIRCLE ALL THAT ARE MENTIONED.**  **IF NONE ARE MENTIONED, SELECT 99.** \| \| NONE-NO CONSEQUENCES/ISSUES \| 1 \| \| --- \| --- \| \| AGAINST THE LAW \| 2 \| \| DENIES BASIC RIGHT TO CHOOSE \| 3 \| \| RISK OF DEATH WITH EARLY PREGNANCY \| 4 \| \| MORE VULNERABLE TO STIs, INCL.HIV \| 5 \| \| MORE LIKELY TO DROP OUT OF SCHOOL \| 6 \| \| MORE VULNERABLE TO ABUSE \| 7 \| \| UNPLANNED PREGNANCY \| 8 \| \| RISK OF MENTAL ILLNESS \| 9 \| \| SOCIAL ISOLATION \| 10 \| \| ECONOMIC DEPENDENCE \| 11 \| \| WORSE NUTRITIONAL OUTCOMES \| 12 \| \| PREFER NOT TO ANSWER \| 66 \| \| DON’T KNOW \| 99 \| \|  \| \| **WI2J.** finally, I would like to ask about your knowledge on forms of violence. Can you name the various forms of VIOLENCE THAT A WOMAN OR FEMALE ADOLESCENT could EXPERIENCE?  **DO NOT PROMPT.** CIRClE ALL RESPONSES THAT ARE SAID.  AFTER, ASK ARE THERE ANY OTHER THINGS? \| **PHYSICAL VIOLENCE**  BEATING/HITTING/KICKING 1  FEMALE GENITAL MUTILATION/INITIATION 2  BURNING 3  **SEXUAL VIOLENCE**  RAPE OR ATTEMPTED RAPE 4  SEXUAL SLAVERY 5  TRAFFICKING 6  SEXUAL HARASSMENT 7  SEXUAL ASSAULT 8  DENIAL OF RIGHT TO USE CONTRACEPTION 9  FORCED ABORTION 10  MOLESTATION 11  FORCED EARLY MARRIAGE 12  NOT DISCLOSING HIV STATUS 13  **EMOTIONAL VIOENCE**  PSYCHOLOGICAL ABUSE 14  THREATS 15  VERBAL ABUSE 16  STALKING 17  **FINANCIAL VIOLENCE**  WITHHOLDING MONEY 18  STEALING 19  PROHIBITING WORKING OR SCHOOL 20  DON’T KNOW 99  PREFER NOT TO ANSWER 66 \|  \|  \| **FAMILY PLANNING MODULE (FP)** \| \| \| \| --- \| --- \| --- \| \| *This section is asked to all women aged 15-49*  I would like to talk with you about another subject – family planning. \| \| \| \| **FP1.** Are you pregnant now? \| Yes, currently pregnant 1  No 2  Prefer not to answer 66  Don’t know 98 \| 2🡪FP3.1  66🡪FP3.1  98🡪FP3.1 \| \| **FP1.1**. How many weeks/months pregnant are you?  Interviewer: write complete months \| Weeks_______________________  Months______________________ \|  \| \| **FP2.** When you got pregnant, did you want to get pregnant at that time? \| Yes 1  No 2  Prefer not to answer 66 \| 1🡪Next module  2🡪FP3.1  66🡪Next module \| \| **FP3.** Did you want to have a baby later on or did you not want any (more) children? \| Later 1  No more/None 2  Prefer not to answer 66 \| 1🡪Next Module  2🡪Next module  66🡪Next module \| \| **FP3.1**. Have you ever HAD a pregnancy that miscarried, or ended in a stillbirth? \| Yes 1  No 2 \|  \| \| **FP3.2.** when did such pregnancy end? \| Weeks-------------------------------  Month----------------------------------  Year____________________________ \|  \| \| 1. When did your last menstrual period start?   **(Date, if given)**  **Month __ __**  **Year __ __ __ __** \| Days ago---------------------------------------1 ___ ___  Weeks ago--------------------------------------2 ___ ___  Months ago-------------------------------------3 ___ ___  Years ago---------------------------------------4 ___ ___  In menopause/has had hysterectomy 994  Before last birth 995  Never menstruated 996  Don’t know 998  Prefer not to answer 966 \| 994-996 🡪Next module \| \| Now I would like to talk about family planning - the various ways or methods that a couple can use to delay or avoid a pregnancy. Have you ever heard of (METHOD)? \| \| \| \| FP4.1. Female Sterilization.  PROBE: Women can have an operation to avoid having any more children. \| Yes 1  No 2 \|  \| \| FP4.2. Male Sterilization.  PROBE: Men can have an operation to avoid having any more children. \| Yes 1  No 2 \|  \| \| FP4.3. IUD.  PROBE: Women can have a loop or coil placed inside them by a  doctor or a nurse which can prevent pregnancy for one or more years. \| Yes 1  No 2 \|  \| \| FP4.4. Injectables.  PROBE: Women can have an injection by a health provider that stops  them from becoming pregnant for one or more months. \| Yes 1  No 2 \|  \| \| FP4.5. Implants.  PROBE: Women can have one or more small rods placed in their  upper arm by a doctor or nurse which can prevent pregnancy for one or more years. \| Yes 1  No 2 \|  \| \| FP4.6. Pill.  PROBE: Women can take a pill every day to avoid becoming pregnant. \| Yes 1  No 2 \|  \| \| FP4.7. Condom.  PROBE: Men can put a rubber sheath on their penis before sexual ntercourse \| Yes 1  No 2 \|  \| \| FP4.8. Female Condom.  PROBE: Women can place a sheath in their vagina before sexual intercourse. \| Yes 1  No 2 \|  \| \| FP4.9. Emergency Contraception.  PROBE: As an emergency measure, within three days after they have  unprotected sexual intercourse, women can take special pills to  prevent pregnancy. \| Yes 1  No 2 \|  \| \| FP4.10. Standard Days Method.  PROBE: A woman uses a string of colored beads to know the days  she can get pregnant. On the days she can get pregnant, she uses a  condom or does not have sexual intercourse. \| Yes 1  No 2 \|  \| \| FP4.11. Lactational Amenorrhea Method (LAM).  PROBE: Up to six months after childbirth, before the menstrual period  has returned, women use a method requiring frequent breastfeeding  day and night. \| Yes 1  No 2 \|  \| \| FP4.12. Rhythm Method.  PROBE: To avoid pregnancy, women do not have sexual intercourse  on the days of the month they think they can get pregnant. \| Yes 1  No 2 \|  \| \| FP4.13. Withdrawal.  PROBE: Men can be careful and pull out before climax. \| Yes 1  No 2 \|  \| \| FP4.13. In the last few months have you:   1. Heard about family planning on the radio? 2. Seen anything about family planning on the television? 3. Read about family planning in a newspaper or magazine? 4. Received a voice or text message about family planning on a mobile phone? 5. Posters/billboards 6. Friends and family members 7. Social media \| Yes NO  radio 1 2  Television 1 2  Newspaper 1 2  Mobile phone 1 2  Posters/billboards 1 2  Friends and family members 1 2  Social media 1 2 \|  \| \| **CHECK FOR PRESENCE OF OTHERS. BEFORE CONTINUING, MAKE EVERY EFFORT TO ENSURE PRIVACY.** \| \| \| \| \| **FP5.** Are you or your partner currently doing something or using any method to delay or avoid getting pregnant? \| Yes 1  No 2  Prefer not to answer 66 \| 2🡪FP7D \| \| 1. What are you currently doing to delay or avoid a pregnancy?   Do not prompt.  If more than one method is mentioned, circle each one. \| Female sterilization A  Male sterilization B  IUD C  Injectables D  Implants E  Pill F  Male condom G  Female condom H  Diaphragm I  Foam / Jelly J  Lactational amenorrhoea method (LAM) K  Periodic abstinence / Rhythm L  Withdrawal M  Standard Days Method………………………….N  Other (*specify*) X  Prefer not to answer Y \|  \| \|  \|  \|  \| \|  \|  \|  \| \|  \|  \|  \| \|  \|  \|  \| \| **FP8A.** Have you ever used anything or tried in any way to delay or avoid getting pregnant in the past? \| Yes 1  No 2  Prefer not to answer 66 \|  \| \| **FP8B.** What have you done to delay or avoid a pregnancy in the past?  Do not prompt.  If more than one method is mentioned, circle each one. \| Female sterilization A  Male sterilization B  IUD C  Injectables D  Implants E  Pill F  Male condom G  Female condom H  Diaphragm I  Foam / Jelly J  Lactational amenorrhoea method (LAM) K  Periodic abstinence / Rhythm L  Withdrawal M  Standard Days Method…………………………..N  Other (*specify*) X  Prefer not to answer Y \|  \| \| \| **CHECK FOR PRESENCE OF OTHERS. BEFORE CONTINUING, MAKE EVERY EFFORT TO ENSURE PRIVACY.** \| \| --- \| \| \| \| \| **FP9A.** Now I have some questions about the future. Would you like to have (a/another) child, or would you prefer not to have any (more) children? \| Have (A/another) child 1  No more/none 2  Says she can’t get pregnant 3  Prefer not to answer 66  Undecided/don’t know 98 \| 2🡪FP9C2  3🡪 Next module  98🡪 Next module \| \| **FP9B.**  How long would you like to wait from now before the birth of (a/another) child? \| Months 1 ___ ___  Years 2 ___ ___  Soon/now 993  Says she can’t get pregnant 994  After marriage 995  Other (*specify*) ____________________996  Don’t know 998  Prefer not to answer 966 \| 993, 994, 998, 966 🡪 END MODULE \| \| **CHECK FP5**:  **NOT CURRENTLY USING** 🡪 **Continue.**  **CURRENTLY USING** 🡪 **END FAMILY PLANNING MODULE.** \| \| \| \| **CHECK FP9B**:  **IF ANSWERED AND WOMAN WANTS A CHILD IN 00-12 MONTHS OR 00-01 YEAR, OR ANSWERED 993,994 OR 998** 🡪 **END FAMILY PLANNING MODULE.**  **ALL ELSE** 🡪 **CONTINUE** \| \| \| \| **CHECK FP9A**:  IF 1🡪 FP9C1  IF 2🡪 FP9C2  **FP9C1. WANTS TO HAVE ANOTHER CHILD:**  "You have said that you do not want (a/another) child **soon**. Can you tell me why you are not using a method to prevent pregnancy?  **DO NOT PROMPT**  **select all mentioned**  Probe: Any other reason?"  **FP9C2. WANTS NO MORE/NONE:**  "You have said that you do not want any (more) children. Can you tell me why you are not using a method to prevent pregnancy?  **DO NOT PROMPT**  **select all mentioned**  Probe: Any other reason?" \| NOT MARRIED/IN A UNION A  **FERTILITY-RELATED REASONS**  NOT HAVING SEX B  INFREQUENT SEX C  MENOPAUSAL/HYSTERECTOMY D  CAN'T GET PREGNANT E  NOT MENSTRUATED SINCE LAST BIRTH F  BREASTFEEDING G  UP TO GOD/FATALISTIC H  **OPPOSITION TO USE**  RESPONDENT OPPOSED I  HUSBAND/PARTNER OPPOSED J  OTHERS OPPOSED K  RELIGIOUS PROHIBITION L  **LACK OF KNOWLEDGE**  KNOWS NO METHOD M  **METHOD-RELATED REASONS**  SIDE EFFECTS/HEALTH CONCERNS N  LACK OF ACCESS/TOO FAR O  COSTS TOO MUCH P  PREFERRED METHOD NOT AVAILABLE Q  NO METHOD AVAILABLE R  INCONVENIENT TO USE S  INTERFERES WITH BODY'S NORMAL PROCESSES T  OTHER (SPECIFY):  ___________________________ X  DON'T KNOW Z  Prefer not to answer Y \|  \|  \| **FERTILITY-SHORT BIRTH HISTORY** \| \| \| \| --- \| --- \| --- \| \| **CM1**. Now I would like to ask about all the births you have had during your life. Have you ever given birth?  *This module should include all births* \| YES 1  NO 2 \| 2*⇨CM8* \| \| **How many babies born alive**  **How many babies stillbirths** \| Alive___________  Stillbirths___________ \|  \| \| 1. what was the month and year of your most recent birth?   I mean the last time you gave birth, even if the child is no longer living, or whose father is not your current partner  **If respondent does not know exact date, probe to estimate month and year** \| Date of last live birth  Month __ __  Year __ __ __ __ \|  \| \| 1. What is the name of this child?   **Record NAME. If child died before being named, write UNNAMED.** \| _________________________________ \|  \| \| 1. Is (NAME) a boy or a girl? \| Boy 1  Girl 2 \|  \| \| **CM2**. Do you have any sons or daughters to whom you have given birth who are now living with you? \| YES 1  NO 2 \| 2*⇨CM5* \| \| **CM3**. How many sons live with you?  *If none, record ‘00’*. \| Sons at home __ __ \|  \| \| **CM4**. How many daughters live with you?  *If none, record ‘00’*. \| Daughters at home __ __ \|  \| \| **CM5**. Do you have any sons or daughters to whom you have given birth who are alive but do not live with you? \| YES 1  NO 2 \| 2*⇨CM8* \| \| **CM6**. How many sons are alive but do not live with you?  *If none, record ‘00’*. \| Sons elsewhere __ __ \|  \| \| **CM7**. How many daughters are alive but do not live with you?  *If none, record ‘00’*. \| Daughters elsewhere __ __ \|  \| \| **CM8**. Have you ever given birth to a boy or girl who was born alive but later died?  If ‘No’ probe by asking:  I mean, to any baby who cried, who made any movement, sound, or effort to breathe, or who showed any other signs of life even if for a very short time? \| YES 1  NO 2 \| 2*⇨CM11* \| \| **CM9**. How many boys have died?  *If none, record ‘00’*. \| Boys dead __ __ \|  \| \| **CM10**. How many girls have died?  *If none, record ‘00’*. \| Girls dead __ __ \|  \| \| **CM11**. Sum answers to CM3, CM4, CM6, CM7, CM9 and CM10. \| Sum __ __ \|  \| \| **CM12**. Just to make sure that I have this right, you have had in total (**total number in CM11**) births during your life. Is this correct? \| Yes 1  No 2 \| 1*⇨CM14* \| \| **CM13**. Check responses to CM1-CM10 and make corrections as necessary until response in CM12 is ‘Yes’. \|  \|  \| \| **CM14**. Check CM11: How many live births? \| No live births, CM11=00 0  One or more live birth,  CM11=01 or more 1 \| 0*⇨End* \|  \| ANTENATAL AND CHILDBIRTH MODULE (CB) \| \|  \| \| --- \| --- \| --- \| \| **These questions should be administered to all women with a live birth in the 2 years preceding date of interview. For women with more than one live birth in the two years preceding the survey, the questions refer only to the most recent live birth.**  **For the interviewer: Check CB1. Did the most recent live birth occur after** **[DATE OF INTERVIEW] 2019?**  **Yes 1🡪Continue to FP7. All questions refer to (NAME) from CB2. Use this child’s name in the following questions, where indicated. If child died before being named, use ‘YOUR BABY’ in place of (NAME)**  **No 2🡪 Skip to Children Under 5 Module** \| \|  \| \| **FP7.** When you got pregnant with (NAME), did you want to get pregnant at that time? \| Yes 1  No 2 \| 1🡪FP9 \| \| **FP8.** Did you want to have a baby later on, or did you not want any (more) children? \| Later 1  No more/None 2 \|  \| \| **FP9.** Has your menstrual period returned since the birth of (NAME)? \| Yes 1  No 2 \|  \| \| 1. While you were pregnant with (*name*) did you see anyone for antenatal care? \| Yes 1  No 2  Don’t know 98 \| 1 🡪CB5B \| \| **CB5A** Why did you not see anyone for antenatal care during your pregnancy with (*name*)? \| Cost too much A  Facility not open B  Too far C  Don’t trust facility D  No female provider at facility E  Husband/family did not allow F  Not necessary G  Not customary H  No transportation I  Poor quality service J  Lack of privacy K  Poor attitude of staff L  Fear of being tested for HIV/AIDS M  Other (*specify*) X \| Skip to CB9 \| \| **CB5B** Whom did you see?  *Probe:* Anyone else?  *Probe for the type of person seen and circle all answers given.* \| Health professional: Doctor/Clinic Officer A  Nurse / Midwife B  Other person  Traditional birth attendant F  Community/ village health worker G  Traditional healer H  Other (*specify*) X \|  \| \| **CB5C** How many weeks or months pregnant were you when you first received antenatal care for this pregnancy?  *Record the answer as stated by respondent.* \| Weeks 1 __ __  Months 2 0 __  DK 98 \|  \| \| 1. How many times did you receive antenatal care when you were pregnant with (NAME)? \| Number of times:__ __  Don’t know 98 \|  \| \| **CB6A.** Did your husband/partner accompany you to any of the antenatal visits? \| Yes 1  No 2 \| Skip if WI2C is 3 \| \| **CB6B.** Did anyone (else) accompany you to any of the antenatal visits? \| Yes 1  No 2 \| 2 🡪CB7A \| \| **CB6C.** Who accompanied you to the antenatal visits?  select all that are mentioned \| Male family member (specify) A  Female family member (specify) B  Other (specify) X \|  \| \| **CB7A** As part of your antenatal care during this pregnancy, were any of the following done at least once:  [A] Was your blood pressure measured?  [B] Did you give a urine sample?  [C] Did you give a blood sample?  [D] I am not interested in knowing your result, but were you given a test for HIV/AIDS? \| Yes No DK  Blood pressure 1 2 98  Urine sample 1 2 98  Blood sample 1 2 98  HIV test 1 2 98 \|  \| \| **CB7B** When you were pregnant with (*name*), did you receive any tetanus injection in the arm or shoulder? \| Yes 1  No 2  DK 98 \|  \| \| **CB7C** How many times did you receive this tetanus injection during your pregnancy with (name)? \| Number of times __  DK 98 \|  \| \| **CB7D** During this pregnancy, were you given or did you buy any iron tablets, iron syrup, or iron and folic acid supplements?  ENUMERATORS TO Show SAMPLE tablets/Syrup TO THE WOMAN \| Yes 1  No 2  DK 98 \|  \| \| **CB7E** During this pregnancy, did you take any drug for intestinal worms? \| Yes 1  No 2  DK 98 \|  \| \| **CB8.** During (any of) your antenatal visit(s) for the pregnancy with (*name*), did you take any medicine in order to prevent you from getting malaria? \| Yes 1  No 2  DK 98 \| 2🡪CB9B  98🡪CB9B \| \| **CB9.** Which medicines did you take to prevent malaria?  *Circle all medicines taken. If type of medicine is not determined, show typical anti-malarial to respondent.* \| SP / Fansidar A  Other (*specify*) X  DK Z \|  \| \| **CB9A.** How many times did you take this malaria medication during this pregnancy \| 1 time 1  2 times 2  3 times 3  4 or more times 4  Don’t know 98 \|  \| \| **CB9A2. How many months pregnant were you when you took your first dose of malaria medication?**  **if don’t know enter “98”** \| Months __ _  Don’t know 98 \|  \| \| Now I would like to ask you about the time when you gave birth to (NAME) \| \|  \| \| **CB9B.** Where did you give birth to (NAME)?  **Only one answer allowed. Probe to identify the type of source.** \| At home 01  On the way to facility 02  Public health sector  Nat. hospital 11  County referral hospital 12  Sub-county hospital 13  Dispensary/maternity centre 14  Health centre 15  Private health sector  Privately owned health facility 21  Faith-based health facility 22  Other (specify) 96 \| 11🡪CB9D  12🡪CB9D  13🡪CB9D  14🡪CB9D  16🡪CB9D  21🡪CB9D  26🡪CB9D  96🡪CB9D \| \| **CB9C.** Why didn’t you deliver (name) in a health facility?  *Probe to identify any other reason*  *Record all mentioned.* \| Cost too much A  Facility not open B  Too far C  Poor attitude of staff D  No female provider at facility E  Lack of privacy F  Husband/family did not allow G  Not necessary H  Not customary I  No transportation J  Poor quality service K  Fear of being tested for HIV/AIDS L  Rapid delivery M  Other (*specify*) X \|  \| \| **CB9D.** Who assisted with the delivery of (NAME)?  **Ask:** “Anyone else?”  **Record all responses. Probe to determine the type of person. If the respondent says that no one assisted, probe to determine whether any adult was present** \| Health professional  Doctor/Clinical Officer 11  Nurse/Midwife 12  Other specify) 16  Other persons  Trained traditional birth attendant 21  Untrained traditional birth attendant 22  Community health worker 23  Relative/friend 24  Self/nobody 25  Traditional healer 26  Other (*specify*) 96  Don’t know 98 \| 21🡪CB9E  22🡪CB9E  24🡪CB9E  ALL ELSE 🡪 CB9F \| \| **CB9E.** What are the reasons you preferred a (Traditional birth attendant/relative) in the birth of (name)?  *Probe to identify any other reason*  *Record all mentioned.* \| Distance A  Better care than facility B  Religious reasons C  Husband/family preference D  Privacy E  Lower costs F  Other (*specify*) X \|  \| \| **CB9F.** Was (*name*) delivered by caesarean section? That is, did they cut your belly open to take the baby out? \| Yes 1  No 2 \|  \| \| **CB9F.1. when was the decision made to have the caesarean section?**  *probe if necessary: was it before or after your labour pains started?* \| Before labour pains…………………………1  After labour pains……………………………2 \|  \| \| **CB9G.** Was your husband/partner present at the facility at the time of the birth? \| Yes 1  No 2 \| Skip if no husband and no facility delivery \| \| **CB9H.** Was anyone (else) present at the facility at the time of the birth? \| Yes 1  No 2 \| skip if no facility \| \| **CB9I.** Who was present?  select all that are mentioned \| Other male member (specify) A  Female family member (specify) B  Other X \|  \| \| **For the interviewer: Check CB8B**  **If 01, 02 or 96 go to CB10D**  **All else continue to CB10A** \| \| \| \| **CB10A.** If delivered in a clinic, overall how satisfied were you with all the services provided during your delivery \| Satisfied 1  Moderately satisfied 2  Dissatisfied 3 \| 2🡪CB10C  3🡪CB10C \| \| **CB10B.** What was the primary reason you were satisfied with the delivery services \| Friendly Staff 1  Good Quality of Care 2  Short Waiting Time 3  Clean/Proper Facility 4  Availability of medicines 5  Availability of equipment/materials (e.g. blankets, etc.) 6  Reasonable Fees 7  Other (specify) 96 \| ALL🡪CB10D \| \| **CB10C.** What is the primary reason you were moderately satisfied or dissatisfied with the delivery services \| Poor Attitude of Staff 1  Poor Quality of Care 2  Long Waiting Time 3  Unclean Facility 4  Unavailability of medicines 5  Unavailability of equipment/materials  (e.g. blankets, etc.) 6  Fees too High 7  Lack of privacy 8  Other (specify) 96 \|  \| \| **CB10D.** Did you or your family make any advance preparations for the birth of (Name) while you were pregnant? \| Yes 1  No 2 \| 2🡪CB10F \| \| **CB10E.** Which of the following preparations did you make?  [A] Discuss the place of delivery?  [B] Discuss who will perform the delivery?  [C] Set aside funds for the delivery?  [D] Make arrangements for transport?  [E] Identify a blood donor?  [G] informed of danger signs of obstetric complications and emergencies  [H] Discuss who will accompany you to planned delivery location  [F] Other preparations (specify) \| Yes No  Place of delivery 1 2  Who delivers 1 2  Set aside funds 1 2  Transport 1 2  Blood donor 1 2  Danger signs 1 2  Company to place of delivery 1 2  Other 1 2 \|  \| \| **FP9.** Has your menstrual period returned since the birth of (NAME)? \| Yes 1  No 2 \|  \| \| **CB10F. Were you asked to bring any supplies to your delivery?**  **Provide examples of supplies: eg. gloves, kanga/clothes, razor, medicine** \| Yes 1  No 2  Don’t know 98 \|  \| \| **CB11A.** Has (NAME) ever been breastfed? \| Yes 1  No 2 \| 2🡪CB11C \| \| **CB11B.** How long after birth did you first put (NAME) to the breast?  **If less than 24 hours, record hours. Otherwise, record days** \| Immediately/within first hour after delivery 00  Hours after birth 1 ___ ___  Days after birth 2___ ___  Don’t know 98 \|  \| \| **CB11C.** Did someone place the baby on your chest, against your skin immediately after delivery? \| Yes 1  No 2  Don’t know 98 \| 2🡪CB11E \| \| **CB11D.** Was your baby wrapped in a towel while lying against your chest or naked against your skin? \| Wrapped in cloth 1  Naked on skin 2  Don’t know 98 \|  \| \| **CB11E.** Was anything applied to the umbilical cord after it was cut?  **Probe:** “Was anything applied to the umbilical cord stump at any time before it separated?” \| Yes 1  No 2  Don’t know 98 \| 2🡪CB11G  98🡪CB11G \| \| **CB11F.** What was applied?  **Probe:** “Anything else?”  **Record all responses** \| Shea butter/oil 01  Ash 02  Mud 03  Dung 04  Breast milk 05  Saliva 06  Herbs/spices 07  Chlorhexidine 08  Other Antiseptic (Alcohol, Spirit) 09  Undetermined 95  Other (specify) 96  Don’t know 98 \|  \| \| **CB11G.** Was (name) dried or wiped after delivery? \| Yes 1  No 2  Don’t know 98 \|  \| \| **CB11H.** How soon after birth was (name) bathed for the first time?  *If less than 1 hour, record “00” hours.* \| Immediately/less than 1 hour 00   Within 12 hours 1  12-24 hours 2  Over 24 hours 3    Never bathed 95    DK / Don’t remember 98 \| 00🡪 CB12  1🡪CB12  2🡪 CB12  95🡪 CB12  998🡪 CB12 \| \| **CB11Ha. How many days after delivery was (name) bathed for the first time** \| Days__ __ \|  \| \| **CB12.** During your pregnancy with (name), did you receive any of the following types of support from your husband/partner?  [A] Ensure you were accompanied to the health facility for antenatal care  [B] Ensure you were accompanied to the health facility for delivery  [C] Purchase necessary items for pregnancy  [D] Support in domestic tasks  [E] Take care of children  [F] Encourage attending ANC visits \| Yes No DK  Ensure accompaniment ANC 1 2 98  Ensure accompaniment delivery 1 2 98  Purchased items 1 2 98  Domestic tasks 1 2 98  Child care 1 2 98  Encouraged ANC 1 2 98 \| Skip if Wi2C is 3 \| \| **CB13.** If not, what was the primary reason why you did not receive support during pregnancy?  Select all mentioned  Probe: any other reasons? \| Husband/ male family member was away A  Childbirth/pregnancy is not for men B  Husband/ male family member does not perceive this is important C  I have other female family members that provide support D  I have my own finances E  I do not require support/help F  Other (Specify) X \| Ask if no to all of CB12 \|  \| Postnatal Care Module (PN) \| \| \| \| --- \| --- \| --- \| \| 1. I would like to talk to you about checks on your health after delivery, for example, someone asking you questions about your health or examining you. Did anyone check on your health after delivery? \| Yes 1  No 2  Don’t know 98 \| 2🡪PN4  98🡪PN4 \| \| **PN1.1**. You have said that you gave birth in (***name or type of facility in CB9B*** ow long did you stay there after the delivery?  *If less than one day, record hours.*  *If less than one week, record days.*  *Otherwise, record weeks.* \| Hours **1** __ __  Days **2** __ __  Weeks **3** __ __  DK/don’t REMEMBER…………………..98 \|  \| \| **PN1A.** As part of the check on your health after delivery, were any of the following done at least once:  [A] Was your blood pressure measured?  [B] Was there a physical examination of the abdomen  [C] Was your temperature measured?  [D] Were you checked for excessive bleeding? \| Yes No DK  Blood pressure 1 2 98  Exam of Abdomen 1 2 98  Temperature 1 2 98  Excessive bleeding 1 2 98 \|  \| \| 1. How long after delivery did your first check take place?   “IF LESS THAN ONE DAY,RECORD HOURS;  IF LESS THAN ONE WEEK, RECORD DAYS." \| Hours **1** __ __  Days **2** __ __  Weeks **3** __ __  DK/don’t REMEMBER…………………..98 \|  \| \| 1. Who checked on your health at that time? \| Health professional  Doctor/Clinical Officer 11  Nurse/Midwife 12  Other specify) 16  Other persons  Trained traditional birth attendant 21  Untrained traditional birth attendant 22  Community health worker 23  Relative/friend 24  Self/nobody 25  Traditional healer 26  Other (*specify*) 96  Don’t know 98 \|  \| \| 1. Now I would like to talk to you about checks on (*name*)’s health after delivery – for example, someone examining (*name*), checking the cord, or seeing if the baby is ok.   After (*name*) was delivered, did anyone check on (*name*)’s health? \| Yes 1  No 2  Don’t know 98 \| 2🡪PN6B  98🡪PN6B \| \| 1. How long after delivery was (NAME)’s health first checked?   “IF LESS THAN ONE DAY,RECORD HOURS;  IF LESS THAN ONE WEEK, RECORD DAYS." \| Hours **1** __ __  Days **2** __ __  Weeks **3** __ __  DK/don’t REMEMBER…………………..98 \|  \| \| 1. Who checked on (*name*)’s health at that time? \| Health professional  Doctor/Clinical Officer 11  Nurse/Midwife 12  Other specify) 16  Other persons  Trained traditional birth attendant 21  Untrained traditional birth attendant 22  Community health worker 23  Relative/friend 24  Self/nobody 25  Traditional healer 26  Other (*specify*) 96  Don’t know 98 \|  \| \| **PN7.** During the check were any of the following done for (name):  [A] Examine the cord?  [B] Counsel you on newborn danger signs?  [C] Assess the temperature of (name)  [D] Counsel you on breastfeeding and observe (name) breastfeeding?  [E] Weigh the baby?  [F] HELP WITH BREASTFEEDING? \| Yes No DK  Examine the cord 1 2 98  Danger signs 1 2 98  Temperature 1 2 98  Breastfeeding counselling 1 2 98  Observe (NAME) breastfeeding 1 2 98  Weigh baby 1 2 98  Help with breastfeeding 1 2 98 \|  \| \| **PN8A.** In the first three days after delivery, was (*name*) given anything to drink other than breast milk? \| Yes 1  No 2  Don’t know 98 \| 2🡪NEXT MODULE  98🡪NEXT MODULE \| \| **PN8B.** What was (*name*) given to drink?  *Probe:* Anything else? \| Milk (other than breast milk) A  Plain water B  Sugar or glucose water C  Gripe water D  Sugar-salt-water solution E  Fruit juice F  Infant formula G  Tea / Infusions H  Honey I  Other (*specify*) X \|  \|  \| DANGER SIGNS – to be asked once to each women with a child under 24 months   \| Now I would like to ask you some questions about danger signs during pregnancy, childbirth and newborn health. \| \| \| \| --- \| --- \| --- \| \| **DW1.** Are you aware of any danger signs during pregnancy? \| Yes 1  No. 2 \| 2🡪DW3 \| \| **DW2.** Could you please name any danger signs during pregnancy that you are aware of?  Select all that are mentioned, **DO NOT READ LIST OR PROMPT** with any suggestions  Probe: Any other danger signs?  *Keep asking for more danger signs until the participant cannot recall any additional signs. Circle all that are mentioned, but do not prompt with any suggestions.* \| Difficulty breathing A  Fatigue/tiredness B  Vaginal bleeding C  Baby not moving D  Loss of consciousness E  Convulsions F  Headache and blurred vision G  Early labour pain before term H  Vaginal discharge I  Signs of malaria  (feeling cold, fever, vomiting) J  High blood pressure K  Swelling of face and arms L  Does not know any of the above M  Other (*specify*) X \|  \|  \| **DW3.** Are you aware of any danger signs during labour and childbirth? \| Yes 1  No. 2 \| 2🡪DW5 \| \| --- \| --- \| --- \| \| **DW4.** Could you please name any danger signs during labour and childrbirth?  Select all that are mentioned, **DO NOT READ LIST OR PROMPT** with any suggestions  Probe: Any other danger signs?  *Keep asking for more danger signs until the participant cannot recall any additional signs. Circle all that are mentioned, but do not prompt with any suggestions.* \| Severe vaginal bleeding (> 12 hours) A  Prolonged labour B  Convulsions C  Retained placenta D  Baby lying sideways or upsidedown E  Fever F  Green or brown waters G  Strong headache H  Blurred or double vision I  Sudden, steady severe pain at the top of the belly J  Overactive reflexes K  High blood pressure L  Protein in the urine M  Does not know any of the above N  Other (*specify*) X \|  \| \| **DW5.** Are you aware any of the danger signs after delivery (for yourself) \| Yes 1  No. 2 \| 2🡪DW7 \| \| **DW6.** What are some of the danger signs that you should watch out for after your delivery?  Select all that are mentioned, **DO NOT READ LIST OR PROMPT** with any suggestions \| High Fever, lower abdominal pain or foul smelling discharge (infection) A  Severe headache, blurred vision, high blood pressure B  Convulsions or fits (eclampsia) C  Heavy vaginal bleeding (PPH) D  Urinary or fecal incontinence (obstetric fistula) E  Extreme tiredness, Anemia F  Anxiety or depression (puerperal psychosis) G  Breast problems (engorgement, sore, cracked bleeding or inverted nipples) H  Does not know any of the above I  Other (*specify*) X \|  \|  \| **DW7.** Are you aware of any danger signs for newborns? \| Yes 1  No. 2 \| 2🡪DW9 \| \| --- \| --- \| --- \| \| **DW8.** Could you please name any danger signs for newborns?  Select all that are mentioned, **DO NOT READ LIST OR PROMPT** with any suggestions  Probe: Any other danger signs?  *Keep asking for more danger signs until the participant cannot recall any additional signs. Circle all that are mentioned, but do not prompt with any suggestions.* \| Convulsions/spasms/rigidity A  Movement only when stimulated or no movement,  even when stimulated B  Not feeding well C  Fever D  Difficult/fast breathing E  Lethargy/unconsciousness F  Yellow or pale color on skin eyes G  Low birth weight H  Not crying I  Does not know any of the above J  Other (*specify*) X \|  \| \| \| --- \| --- \| --- \| --- \| --- \| --- \| --- \| --- \| --- \| --- \| --- \| --- \| --- \| --- \| --- \| --- \| --- \| --- \| --- \| --- \| --- \| --- \| --- \| --- \| --- \| --- \| --- \| --- \|  \| **SECTION 3: questionnaire for children under five** \| \| \| --- \| --- \| \|  \| \| \| **under-five child information panel uf** \| \| \| *This questionnaire is to be administered to all mothers or caretakers (see List of Household Members, column HL8-10) who care for a child that lives with them and is under the age of 5 years (see List of Household Members, column HL7B).*  *A separate questionnaire should be used for each eligible child.* \| \| \| UF1. Cluster number: \| UF2. Household number: \| \| ___ ___ ___ \| ___ ___ \| \| UF3. Child’s name: Name (use this NAME, where indicated in the questionnaire): \| UF4. Child’s line number: \| \| Name \| ___ ___ \| \| UF5. Mother’s / Caretaker’s name: \| UF6. Mother’s / Caretaker’s line number: \| \| Name \| ___ ___ \| \| **UF7. CHECK:** Has the woman’s consent/assent form already been administered to the mother or caretaker?  Yes 1 🡪Complete date, interviewer name, number and result for the appropriate Visit.  **Do not re-administer consent/assent script**  No 2🡪 UF8 \| \| \| UF8. Check HH6 and record woman’s age: ___ ___ \| \| \| UF9. Is woman/caregiver married?  Check WI2C  Yes 1  No--------------------------------------------------------2 \| \| \| **If the woman is 15-17 years old and unmarried, read the consent script to the woman’s mother, father or caretaker and the assent script to the woman. You MUST obtain the consent of the person the woman is comfortable with before proceeding**  **If the woman is married, or is unmarried and older than 17 years, read her the woman’s consent script. You MUST obtain the woman’s consent before proceeding**  **If consent/assent script has already been administered to the mother or caretaker, do not re-administer the script** \| \| \| UF10. Interviewer’s name and number: \| UF11. Day / Month / Year of interview: \| \| Name ___ ___ \| ___ ___ /___ ___ / 2 0 1 ___ \|  \| *Repeat greeting if not already read to this respondent:*  We are from ***insert country-specific affiliation***. We are conducting a survey about the situation of children, families and households. I would like to talk to you about (*child’s name from CB3*)’s health and well-being. The interview will take about ***insert number*** minutes. All the information we obtain will remain strictly confidential and anonymous. \| *If greeting at the beginning of the household questionnaire has already been read to this person, then read the following:*  Now I would like to talk to you more about (*child’s name from CB3*)’s health and other topics. This interview will take about ***insert number*** minutes. Again, all the information we obtain will remain strictly confidential and anonymous. \| \| --- \| --- \| \| May I start now?   - *Yes, permission is given* ⇨ *Begin the interview.* - *No, permission is not given* ⇨  *Circle ‘03’ in UF12. Discuss this result with your supervisor.* \| \|  \| UF12. Result of interview for children under 5  *Codes refer to mother/caretaker.* \| Completed 01  Not at home 02  Not competent 03  Deferred 04  Refused 05  Partially completed 06  Other (*specify*) 96 \| \| --- \| --- \|  \| CHILD INFORMATION (CI) \| \| \| \| --- \| --- \| --- \| \| 1. Now I would like to ask you some questions about the health of (*name*).   On what day, month and year was (*name*) born?  *Probe:*  What is his / her birthday?  *If the mother/caretaker knows the exact birth date, also enter the day; otherwise, circle 98 for day.*  *Month and year must be recorded.* \| Date of birth Day __ __  DK day 98  Month __ __  Year 2 0 __ __ \|  \| \| 1. How old is (*name*)?   *Probe*:  How old was (*name*) at his / her last birthday?  **If If less than 1 year, record in completed months, otherwise record in completed years.**  **Compare and correct CI1 and CI2 if inconsistent** \| Age (in years if greater than 1 year) _________  Age (in months if less than 1 year) _________ \|  \| \| 1. Is (NAME) a boy or a girl? \| Boy 1  Girl 2 \|  \|  \| **BREASTFEEDING AND NUTRITION** \| \| \| \| \| \| \| \| \| \| \| --- \| --- \| --- \| --- \| --- \| --- \| --- \| --- \| --- \| --- \| \| **THIS MODULE SHOULD BE ADMINISTERED TO MOTHERS OR PRIMARY CARETAKER OF CHILDREN AGED LESS THAN 2 YEARS, FOR EACH CHILD. CHECK CI1. IF (NAME) WAS BORN BEFORE [INTERVIEW DATE] 2014, GO TO THE IMMUNIZATION MODULE (IM)** \| \| \| \| \| \| \| \| \| \| \| *Check HL6: Household Member Age*  **🞎**  *Child age 0, 1 or 2 ⇨ Continue with BN1.*  **🞎** Child age 3 or 4 ⇨ Next Module  Now I want to ask you about how (NAME) is fed \| \| \| \| \| \| \| \| \| \| \| 1. Has (*name*) ever been breastfed? \| \| \| Yes 1  No 2  Don’t know 98 \| \| \| \| \| 8 🡪 BN3  8 🡪 BN3 \| \| \| 1. Is (*name*) still being breastfed? \| \| \| Yes 1  No 2  Don’t know 98 \| \| \| \| \| 1🡪 BN3 \| \| \| **BN2A.** For how many months  did you breastfeed (*name*)? \| \| \| Months _____ ______  Don’t know 98 \| \| \| \| \|  \| \| \| **BN3.** Now I would like to ask you about the liquids or foods that (NAME) had yesterday during the day or at night. I am interested in whether (NAME) had the item I mention, even if it was combined with other foods.  Please include liquids or foods consumed outside of your home.  Did (NAME) drink/eat:  **Read each item aloud and record the response before proceeding to the next item. Record YES if the child consumed the liquid or food alone or mixed with other liquids or foods** \| \| \| \| \| \| \|  \| \| \|  \| \|  \| Yes \| No \| DK \| \|  \| \| \| [A] Breastmilk? \| \| Breastmilk \| 1 \| 2 \| 98 \| \|  \| \| \| [B] Plain water? \| \| Plain water \| 1 \| 2 \| 98 \| \|  \| \| \| [C] Medicine or ORS \| \| Medicine or ORS \|  \|  \|  \| \|  \| \| \| [D] Juice or juice drinks? \| \| Juice or juice drinks \| 1 \| 2 \| 98 \| \|  \| \| \| [E] ***insert local name for clear broth / clear soup***? (Note: to be added during the training with the enumerators in collaboration with the country teams) \| \| Soup \| 1 \| 2 \| 98 \| \|  \| \| \| [F] Milk such as tinned, powdered, or fresh animal milk? \| \| Milk \| 1 \| 2 \| 98 \| \|  \| \| \| - 1. *If yes:* How many times did (*name)* drink milk? *If 7 or more times, record '7'*.   2. *If unknown, record ‘8’.* \| \| Number of times drank milk __ \| \| \| \| \|  \| \| \| [G] Infant formula? \| \| Infant formula \| 1 \| 2 \| 98 \| \|  \| \| \| 1. *If yes:* How many times did (*name)* drink infant formula? 2. *If 7 or more times, record '7'*. 3. *If unknown, record ‘8’.* \| \| Number of times drank infant formula __ \| \| \| \| \|  \| \| \| [H] Any other liquids?   1. (*Specify*)_____________________________ \| \| Other liquids \| 1 \| 2 \| 98 \| \|  \| \| \| [I] Yogurt? \| \| Yogurt \| 1 \| 2 \| 98 \| \|  \| \| [J] “Any nestle, cerelac, nestum, farex \| \| Cerelac \| 1 \| 2 \| 98 \| \|  \| \| [K] Bread, rice, noodles, porridge, mandazi, chapati or other foods made from grains? \| \| Foods made from grains \| 1 \| 2 \| 98 \| \|  \| \| [L] Pumpkin, carrots, squash or sweet potatoes that are yellow or orange inside? \| \| Pumpkin, carrots, squash, etc. \| 1 \| 2 \| 98 \| \|  \| \| [M] White potatoes, white yams, white sweet potato, cassava, matoka or any other foods made from roots? \| \| White potatoes, white yams, white sweet potato, cassava, etc. \| 1 \| 2 \| 98 \| \|  \| \| [N] Any dark green, leafy vegetables? \| \| Dark green, leafy vegetables \| 1 \| 2 \| 98 \| \|  \| \| [O] Ripe mangoes, papayas or ***insert any other locally available vitamin A-rich fruits***?(Note: to be added during the training with the enumerators in collaboration with the country teams) \| \| Ripe mangoes \| 1 \| 2 \| 98 \| \|  \| \| [P] Any other fruits or vegetables? \| \| Other fruits or vegetables \| 1 \| 2 \| 98 \| \|  \| \| [Q] Liver, kidney, heart or other organ meats? \| \| Liver, kidney, heart or other organ meats \| 1 \| 2 \| 98 \| \|  \| \| [R] Any meat, such as beef, pork, lamb, goat, chicken, or duck? \| \| Meat, such as beef, pork, lamb, goat, etc. \| 1 \| 2 \| 98 \| \|  \| \| [S] Eggs? \| \| Eggs \| 1 \| 2 \| 98 \| \|  \| \| [T] Fresh or dried fish or shellfish? \| \| Fresh or dried fish \| 1 \| 2 \| 98 \| \|  \| \| [U] Any foods made from beans, peas, lentils, or nuts? \| \| Foods made from beans, peas, etc. \| 1 \| 2 \| 98 \| \|  \| \| [V] Cheese or other food made from milk? \| \| Cheese or other food made from milk \| 1 \| 2 \| 98 \| \|  \| \| [W] Any other solid, semi-solid, or soft food that I have not mentioned?  (*Specify*)_____________________________ \| \| Other solid, semi-solid, or soft food \| 1 \| 2 \| 98 \| \|  \| \| **BN4***. Check BN3 (Categories “G” through “W”).*  **🞎** *At least one “Yes” or all “DK” ⇨ Go to BN6.*  **🞎** *Else ⇨ Continue with BN5.* \| \| \| \| \| \| \| \| \| **BN5. "**Did (NAME) eat any solid, semi-solid, or soft foods yesterday during the day or at night?  IF ‘YES’ PROBE: What kind of solid, semi-solid or soft foods did (NAME) eat?"  AND record response in BN3 \| Yes 1  No 2 \| \| \| \| \| 2🡪Next module \| \| \| **BN6.** How many times did (*name*) eat any solid, semi-solid or soft foods yesterday during the day or night?  *If 7 or more times, record '7'*. \| Number of times __  Don’t know 98 \| \| \| \| \|  \| \| \| **BN6A.** In the last 7 days, was (name) given iron pills, sprinkles with iron, or iron syrup like (this/any of these)?  Show common types of pills/sprinkles/syrups \| Yes 1  No 2  Don’t know 98 \| \| \| \| \|  \| \| \| The below question should only be asked **once** to each mother/caregiver of a child under 2 \| \| \| \| \| \| \| \| \| Now I would like to ask you about your awareness of child nutrition practices \| \| \| \| \| \| \| \| \| **BN6B**. What are the recommended ways of feeding newborns and children under two years of age?  *.*  Select all that are mentioned, **DO NOT READ LIST OR PROMPT** with any suggestions  Probe: Any other recommended ways?  *Keep asking for more recommended ways until the participant cannot recall any more.* \| Early initiation of breastfeeding (within one hour) A  Exclusive breastfeeding for the first six months B  Nutritionally adequate and safe complementary  foods after 6 complete months C  Continued breastfeeding for up to two years  of age or beyond D  Does not know any of the above X \| \| \| \| \|  \| \|  \| **immunization IM** \| \| \| \| \| \| \| \| \| \| \| \| --- \| --- \| --- \| --- \| --- \| --- \| --- \| --- \| --- \| --- \| --- \| \| *This section is to be administered to all mothers or primary caregiver of children age 0-2 years.*  *If an immunization* ***(child health)*** *card is available, copy the dates in IM2 for each type of immunization recorded on the card. IM4-IM4C will only be asked if a card is not available.* \| \| \| \| \| \| \| \| \| \| \| \| 1. Do you have a card where (*name*)’s vaccinations are written down?   *If yes:* May I see it please? \| \| Yes, observed immunization booklet/card 1  Yes, not observed booklet/card 2  No card 3 \| \| \| \| \| \| \| \|  \| \| **IM1A.** Do you have a MCH booklet?  *If yes:* May I see it please? \| \| Yes, observed MCH booklet 1  Yes, not observed booklet 2  No Booklet 3 \| \| \| \| \| \| \| \|  \| \| **If IM1 is 1 or IM1A is 1 IM2** 🡪 IM2  **if neither IM1 or IM1A is 1** 🡪 IM4 \| \| \| \| \| \| \| \| \| \| \| \| 1. *(a) Copy dates for each vaccination from the card.*   *(b) Write ‘44’ in day column if card shows that vaccination was given but no date recorded.* \| \| Date of Immunization \| \| \| \| \| \| \| \|  \| \| Day \| \| Month \| \| Year \| \| \| \| \| **Pentavalent/DPT 1** \|  \|  \|  \|  \|  \|  \|  \|  \|  \|  \| \| **Pentavalent/DPT 2** \|  \|  \|  \|  \|  \|  \|  \|  \|  \|  \| \| **Pentavalent/DPT 3** \|  \|  \|  \|  \|  \|  \|  \|  \|  \|  \| \| **Measles (or MMR or MR)** \|  \|  \|  \|  \|  \|  \|  \|  \|  \|  \| \| *Check IM2. Are all vaccines recorded?*  **🞎** *Yes ⇨ Go to Next Module.*  **🞎** *No ⇨ Continue with IM3.* \| \| \| \| \| \| \| \| \| \| \| \| 1. Has (*name*) ever received any vaccinations in im2 that are not recorded on this card, including vaccinations received in campaigns or immunization days or child health days?   **🞎** *Yes ⇨ Go back to IM2 and probe for these vaccinations and write ‘66’ in the corresponding day column   for each vaccine mentioned. When finished, skip to Next module.*  **🞎** *No/DK ⇨ End and go to Care Seeking Module* \| \| \| \| \| \| \| \| \| \| \| \| 1. Has (*name*) ever received any vaccinations to prevent him/her from getting diseases, including vaccinations received in a campaign or immunization day or child health day? \| \| Yes 1  No 2  Don’t know 98 \| \| \| \| \| \| \| \| 2**🡪**END  98**🡪**END \| \| **IM4A.** Has (NAME) ever received a pentavalent vaccination, that is, an injection given in the thigh sometimes at the same time as polio drops? \| \| Yes 1  No. 2  Don’t know 98 \| \| \| \| \| \| \| \| 2🡪W48  98🡪W48 \| \| **IM4B.** How many times was a Pentavalent/DPT vaccine received? \| \| Number of times ____  Don’t know 98 \| \| \| \| \| \| \| \|  \| \| **IM4C.** Has (name) ever received a Measles injection (or an MMR or MR) – that is, a shot in the arm at the age of 9 months or older - to prevent him/her from getting measles? \| \| Yes 1  No. 2  Don’t know 98 \| \| \| \| \| \| \| \|  \|  \| **VITAMIN A MODULE (VA)** \| \| \| \| --- \| --- \| --- \| \| ***To be completed for all children 0-59 months*** \| \| \| \| **VA1A.** Has (NAME) received 2 vitamin A doses like this one in the past year?  **Show capsules** \| Yes 1  No 2  Don’t know 98 \|  \|  \| **COUGH AND FEVER MODULE (CO)** \| \| \| \| --- \| --- \| --- \| \| 1. Has (Name) been ill with a fever at any time in the last 2 weeks? \| Yes 1  No 2  Don’t know 98 \| 2🡪CO2  98🡪CO2 \| \| **CO1A.** At any time during the illness, did (*name*) have blood taken from his/her finger or heel for testing? \| Yes 1  No 2  Don’t know 98 \|  \| \| 1. Has (name) been ill with a cough at any time in the last 2 weeks? \| Yes 1  No 2  Don’t know 98 \| 2🡪CO5  98🡪CO5 \| \| 1. When (*name*) was ill with a cough, did he/she breathe faster than usual with short, fast breaths or did he/she have difficulty breathing? \| Yes 1  No 2  Don’t know 98 \| 2🡪CO5  8🡪CO5 \| \| 1. Was the difficult breathing due to a problem in the chest or a blocked or runny nose? \| Problem in chest only 1  Blocked or runny nose only 2  Both 3  Other (*specify*) 6  Don’t know 98 \|  \| \| 1. *Check CO1 for fever and C02 for cough*   **🞎** *Child had fever and/or cough* ⇨ *Continue with CO6*  **🞎** *No or DK for fever AND no or DK for cough ⇨ Next module.* \| \| \| \| 1. Did you seek advice or treatment for the illness from any source? \| Yes 1  No 2  Don’t know 98 \| 2🡪CO8  98🡪CO8 \| \| 1. Where did you seek advice or treatment?   **DO NOT PROMPT**  *Probe:* Anywhere else?  *Circle all providers mentioned,*  *but do NOT prompt with any suggestions.*  *If unable to determine write the name of the place.*  *Probe to identify each type of source.* \| Public health sector  County referral hospital 12  Sub-county hospital 13  Dispensary/maternity centre 14  Health centre 15  Private health sector  Privately owned health facility 21  Faith-based health facility 22  Pharmacy 23  Mobile clinic 24  Community  Community health worker 31  Informal drug seller 32  Shop 33  Traditional healer 34  Other (specify) 96 \|  \| \| **CO7A.** Why didn’t you seek advice or treatment from a medical provider?  *Probe to identify any other reason*  *Record all mentioned.* \| Cost too much A  Facility not open B  Too far C  Don’t trust facility D  Not necessary E  Husband/family did not allow F  No transportation G  Poor quality service H  Other (*specify*) X \|  \| \| 1. At any time during the illness, did (*name*) take any drugs for the illness? \| Yes 1  No 2  Don’t know 98 \| 2🡪NEXT MODULE  98🡪NEXT MODULE \| \| 1. What drugs did (*name*) take?   *Probe:*  Any other medicine?  *Circle all medicines given. Write brand name(s) of all medicines mentioned.* \| Anti-malarials:  SP / Fansidar A    Amodiaquine B  Quinine C  Combination with Artemisinin (ACT) D  Other anti-malarial  (*specify*) E  Antibiotics:  Pill / Syrup H  Injection I  Other medications:  Paracetamol/ Panadol /Acetaminophen J  Aspirin K  Ibuprofen L  Other (*specify*) X  DK Z \|  \| \| ***CO9A.*** *Check CO9: Antibiotic mentioned (codes I or J)?*  **🞎***Yes* ⇨ *Continue with CO9B.*  **🞎** *No* ⇨ *Go to CO9C.* \| \| \| \| **CO9B.** Where did you get the (*name of medicine from CO9*)?  **DO NOT PROMPT**  *Probe to identify the type of source.*  *If unable to determine write the name of the place.* \| Public health sector  County referral hospital 12  Sub-county hospital 13  Dispensary/maternity centre 14  Health centre 15  Private health sector  Privately owned health facility 21  Faith-based health facility 22  Pharmacy 23  Mobile clinic 24  Community  Community health worker 31  Informal drug seller 32  Shop 33  Traditional healer 34  Other (specify) 96 \|  \| \| ***CO9C.*** *Check CO9: Anti-malarial mentioned (codes A - H)?*  **🞎** *Yes* ⇨ *Continue with CO9D.*  **🞎** *No* ⇨ *NEXT MODULE* \| \| \| \| **CO9D.** Where did you get the (*name of medicine from CO9*)?  *Probe to identify the type of source.* \| Public health sector  Nat. hospital 11  Reg. hospital 12  Dispensary/maternity 13  Health center 14  Other public (specify) 16  Private health sector  Private hospital/clinic 21  Pharmacy 22  Mobile clinic 23  Other private (specify) 26  Community  Community health worker 31  Informal drug seller 32  Shop 33  Traditional healer 34  Other community(specify) 36 \|  \| \| **CO9E.** How long after the fever started did (*name*) first take (*name of anti-malarial from CO9D)*?  *If multiple anti-malarials mentioned in CA25, name all anti-malarial medicines mentioned.* \| Same day 0  Next day 1  2 days after the fever 2  3 days after the fever 3  4 or more days after the fever 4  Don’t know 98 \|  \|  \| **D****IARRHEA MODULE (DI) DI** \| \| \| \| --- \| --- \| --- \| \| *This section is to be administered to all mothers or caretakers of children under the age of 5 years.* \| \| \| \| **DI1.** Has (NAME) had diarrhoea in the last 2 weeks?  *Diarrhoea is defined as three or more loose or watery stools per day, or blood in stool. If child is exclusively breastfeeding, diarrhea is determined as perceived by mother or caretaker.* \| Yes 1  No 2  Don’t know 98 \| 2🡪DI Next Module  98🡪 Next Module \| \| **DI1A.** I would like to know how much (*name*) was given to drink during the diarrhoea (including breastmilk).  During the time (*name*) had diarrhoea, was he/she given less than usual to drink, about the same amount, or more than usual?  *If ‘less’, probe*:  Was he/she given much less than usual to drink, or somewhat less? \| Much less 1  Somewhat less 2  About the same 3  More 4  Nothing to drink 5  Don’t know 98 \|  \| \| **DI1B**. During the time (*name*) had diarrhoea, was he/she given less than usual to eat, about the same amount, more than usual, or nothing to eat?  *If ‘less’, probe:*  Was he/she given much less than usual to eat or somewhat less? \| Much less 1  Somewhat less 2  About the same 3  More 4  Stopped food 5  Never gave food 6  Don’t know 98 \|  \| \| **DI1C**. Did you seek any advice or treatment for the diarrhoea from any source? \| Yes 1  No 2  Don’t know 98 \| 2🡪DI1E  98🡪DI1E \| \| **DI1D**. From where did you seek advice or treatment?  *Probe:*  Anywhere else?  *Circle all providers mentioned,*  *but do NOT prompt with any suggestions.*  *Probe to identify each type of source.* \| Public sector  County referral hospital A  Sub-county hospital B  Government health centre C  Community health worker D  Mobile / Outreach clinic E  Private medical sector  Private hospital / clinic G  Private physician H  Private pharmacy I  Mobile clinic J  Other source  Relative / Friend K  Shop L  Traditional healer M  Other (*specify*) X \| ALL🡪DI2 \| \| **DI1E**. Why did you not seek advice or treatment?  *Probe to identify any other reason*  *Record all mentioned.* \| Episode was not serious A  Too far/no transport B  Too expensive C  Believe home remedies are effective D  Husband/family did not allow E  No reason F  Other (specify) X \|  \| \| **DI2**. When (NAME) had the diarrhea in the past 2 weeks, was he/she ever given a fluid prepared from a packet called Orasel or ORS (oral rehydration solution)?  **Show local ORS sachets** **or fluid** [B] \| Yes 1  No 2  Don’t know 98 \|  \| \| **DI2A.** *Check DI2: ORS.*  **🞎**  *Child was given ORS (‘Yes’ in DI2) ⇨ Continue with DI2B.*  **🞎**  *Child was not given ORS ⇨ Go to DI2C.* \| \| \| \| **DI2B.** Where did you get the ORS?  *Probe to identify the type of source.* \| Public health sector  County referral hospital 11  Sub-county hospital 12  Dispensary/maternity 13  Health center 14  Private health sector  Privately owned health facility 21  Faith-based health facility 22  Pharmacy 23  Mobile clinic 24  Community  Community health worker 31  Informal drug seller 32  Shop 33  Traditional healer 34  Other (specify) 96 \|  \| \| **DI2C.** During the time (*name*) had diarrhoea, was (*name*) given:  [A] zinc tablets?  [B] zinc syrup? \| Y N DK  Zinc tablets 1 2 98  Zinc syrup 1 2 98 \|  \| \| **DI2D.** *Check DI2C: Any zinc?*  **🞎**  *Child given any zinc (‘Yes’ circled in ‘A’ or ‘B’ in DI2C) ⇨ Continue with DI2E.*  **🞎**  *Child was not given any zinc ⇨ Go to DI3.* \| \| \| \| **DI2E.** Where did you get the zinc?  *Probe to identify the type of source.* \| Public health sector  County referral hospital 11  Sub-county hospital 12  Dispensary/maternity 13  Health center 14  Private health sector  Privately owned health facility 21  Faith-based health facility 22  Pharmacy 23  Mobile clinic 24  Community  Community health worker 31  Informal drug seller 32  Shop 33  Traditional healer 34  Other (specify) 96 \|  \| \| **DI3.** Was anything (else) given to treat the diarrhoea? \| Yes 1  No 2  Don’t know 98 \| 2🡪NEXT MODULE  98🡪NEXT MODULE \| \| **DI4.** What (else) was given to treat the diarrhoea?  *Probe*:  Anything else?  *Record all treatments given.* \| Pill or syrup  Antibiotic A  Antimotility B  Other (specify) C  Unknown pill or syrup D  Injection  Antibiotic E  Other (specify) F  Unknown injection G  Intravenous (IV) H  Traditional/home remedy I  Other (specify) X \|  \|  \| \| CHILD DANGER SIGNS MODULE (DC)  To be asked **once** to each women with a child under 5 years \| \| \| --- \| --- \| \| **DC1.** Sometimes children have severe illness and should be taken to a health facility. What types of symptoms would cause you to take a child under the age of 5 to a health facility right away?  Select all that are mentioned, **DO NOT READ LIST OR PROMPT** with any suggestions  Probe: Any other symptoms?  *Keep asking for more symptoms until the participant cannot recall any more.* \| Child not able to drink or breastfeed A  Child becomes sicker B  Child develops a fever C  Child has fast breathing D  Child has difficulty breathing E  Child has blood in stool F  Child is drinking poorly G  Child is vomiting H  Child is lethargic I  Child is unconscious J  Child doesn’t cry K  Child’s skin is yellow L  Does not know any of the above M  Other (*specify*) X \| \| **DC2.** When a child has diarrhea, how much should he or she be given to drink; more than usual, the same as usual, less than usual, or should he or she not be given anything to drink at all? \| More than usual 1  About the same 2  Less than usual 3  Nothing to drink 4  Don’t know 98 \| \| \| --- \| --- \| --- \| --- \| --- \| --- \| --- \| |
| --- | --- | --- | --- | --- | --- | --- | --- | --- | --- | --- | --- | --- | --- | --- | --- | --- | --- | --- | --- | --- | --- | --- | --- | --- | --- | --- | --- | --- | --- | --- | --- | --- | --- | --- | --- | --- | --- | --- | --- | --- | --- | --- | --- | --- | --- | --- | --- | --- | --- | --- | --- | --- | --- | --- | --- | --- | --- | --- | --- | --- | --- | --- | --- | --- | --- | --- | --- | --- | --- | --- | --- | --- | --- | --- | --- | --- | --- | --- | --- | --- | --- | --- | --- | --- | --- | --- | --- | --- | --- | --- | --- | --- | --- | --- | --- | --- | --- | --- | --- | --- | --- | --- | --- | --- | --- | --- | --- | --- | --- | --- | --- | --- | --- | --- | --- | --- | --- | --- | --- | --- | --- | --- | --- | --- | --- | --- | --- | --- | --- | --- | --- | --- | --- | --- | --- | --- | --- | --- | --- | --- | --- | --- | --- | --- | --- | --- | --- | --- | --- | --- | --- | --- | --- | --- | --- | --- | --- | --- | --- | --- | --- | --- | --- | --- | --- | --- | --- | --- | --- | --- | --- | --- | --- | --- | --- | --- | --- | --- | --- | --- | --- | --- | --- | --- | --- | --- | --- | --- | --- | --- | --- | --- | --- | --- | --- | --- | --- | --- | --- | --- | --- | --- | --- | --- | --- | --- | --- | --- | --- | --- | --- | --- | --- | --- | --- | --- | --- | --- | --- | --- | --- | --- | --- | --- | --- | --- | --- | --- | --- | --- | --- | --- | --- | --- | --- | --- | --- | --- | --- | --- | --- | --- | --- | --- | --- | --- | --- | --- | --- | --- | --- | --- | --- | --- | --- | --- | --- | --- | --- | --- | --- | --- | --- | --- | --- | --- | --- | --- | --- | --- | --- | --- | --- | --- | --- | --- | --- | --- | --- | --- | --- | --- | --- | --- | --- | --- | --- | --- | --- | --- | --- | --- | --- | --- | --- | --- | --- | --- | --- | --- | --- | --- | --- | --- | --- | --- | --- | --- | --- | --- | --- | --- | --- | --- | --- | --- | --- | --- | --- | --- | --- | --- | --- | --- | --- | --- | --- | --- | --- | --- | --- | --- | --- | --- | --- | --- | --- | --- | --- | --- | --- | --- | --- | --- | --- | --- | --- | --- | --- | --- | --- | --- | --- | --- | --- | --- | --- | --- | --- | --- | --- | --- | --- | --- | --- | --- | --- | --- | --- | --- | --- | --- | --- | --- | --- | --- | --- | --- | --- | --- | --- | --- | --- | --- | --- | --- | --- | --- | --- | --- | --- | --- | --- | --- | --- | --- | --- | --- | --- | --- | --- | --- | --- | --- | --- | --- | --- | --- | --- | --- | --- | --- | --- | --- | --- | --- | --- | --- | --- | --- | --- | --- | --- | --- | --- | --- | --- | --- | --- | --- | --- | --- | --- | --- | --- | --- | --- | --- | --- | --- | --- | --- | --- | --- | --- | --- | --- | --- | --- | --- | --- | --- | --- | --- | --- | --- | --- | --- | --- | --- | --- | --- | --- | --- | --- | --- | --- | --- | --- | --- | --- | --- | --- | --- | --- | --- | --- | --- | --- | --- | --- | --- | --- | --- | --- | --- | --- | --- | --- | --- | --- | --- | --- | --- | --- | --- | --- | --- | --- | --- | --- | --- | --- | --- | --- | --- | --- | --- | --- | --- | --- | --- | --- | --- | --- | --- | --- | --- | --- | --- | --- | --- | --- | --- | --- | --- | --- | --- | --- | --- | --- | --- | --- | --- | --- | --- | --- | --- | --- | --- | --- | --- | --- | --- | --- | --- | --- | --- | --- | --- | --- | --- | --- | --- | --- | --- | --- | --- | --- | --- | --- | --- | --- | --- | --- | --- | --- | --- | --- | --- | --- | --- | --- | --- | --- | --- | --- | --- | --- | --- | --- | --- | --- | --- | --- | --- | --- | --- | --- | --- | --- | --- | --- | --- | --- | --- | --- | --- | --- | --- | --- | --- | --- | --- | --- | --- | --- | --- | --- | --- | --- | --- | --- | --- | --- | --- | --- | --- | --- | --- | --- | --- | --- | --- | --- | --- | --- | --- | --- | --- | --- | --- | --- | --- | --- | --- | --- | --- | --- | --- | --- | --- | --- | --- | --- | --- | --- | --- | --- | --- | --- | --- | --- | --- | --- | --- | --- | --- | --- | --- | --- | --- | --- | --- | --- | --- | --- | --- | --- | --- | --- | --- | --- | --- | --- | --- | --- | --- | --- | --- | --- | --- | --- | --- | --- | --- | --- | --- | --- | --- | --- | --- | --- | --- | --- | --- | --- | --- | --- | --- | --- | --- | --- | --- | --- | --- | --- | --- | --- | --- | --- | --- | --- | --- | --- | --- | --- | --- | --- | --- | --- | --- | --- | --- | --- | --- | --- | --- | --- | --- | --- | --- | --- | --- | --- | --- | --- | --- | --- | --- | --- | --- | --- | --- | --- | --- | --- | --- | --- | --- | --- | --- | --- | --- | --- | --- | --- | --- | --- | --- | --- | --- | --- | --- | --- | --- | --- | --- | --- | --- | --- | --- | --- | --- | --- | --- | --- | --- | --- | --- | --- | --- | --- | --- | --- | --- | --- | --- | --- | --- | --- | --- | --- | --- | --- | --- | --- | --- | --- | --- | --- | --- | --- | --- | --- | --- | --- | --- | --- | --- | --- | --- | --- | --- | --- | --- | --- | --- | --- | --- | --- | --- | --- | --- | --- | --- | --- | --- | --- | --- | --- | --- | --- | --- | --- | --- | --- | --- | --- | --- | --- | --- | --- | --- | --- | --- | --- | --- | --- | --- | --- | --- | --- | --- | --- | --- | --- | --- | --- | --- | --- | --- | --- | --- | --- | --- | --- | --- | --- | --- | --- | --- | --- | --- | --- | --- | --- | --- | --- | --- | --- | --- | --- | --- | --- | --- | --- | --- | --- | --- | --- | --- | --- | --- | --- | --- | --- | --- | --- | --- | --- | --- | --- | --- | --- | --- | --- | --- | --- | --- | --- | --- | --- | --- | --- | --- | --- | --- | --- | --- | --- | --- | --- | --- | --- | --- | --- | --- | --- | --- | --- | --- | --- | --- | --- | --- | --- | --- | --- | --- | --- | --- | --- | --- | --- | --- | --- | --- | --- | --- | --- | --- | --- | --- | --- | --- | --- | --- | --- | --- | --- | --- | --- | --- | --- | --- | --- | --- | --- | --- | --- | --- | --- | --- | --- | --- | --- | --- | --- | --- | --- | --- | --- | --- | --- | --- | --- | --- | --- | --- | --- | --- | --- | --- | --- | --- | --- | --- | --- | --- | --- | --- | --- | --- | --- | --- | --- | --- | --- | --- | --- | --- | --- | --- | --- | --- | --- | --- | --- | --- | --- | --- | --- | --- | --- | --- | --- | --- | --- | --- | --- | --- | --- | --- | --- | --- | --- | --- | --- | --- | --- | --- | --- | --- | --- | --- | --- | --- | --- | --- | --- | --- | --- | --- | --- | --- | --- | --- | --- | --- | --- | --- | --- | --- | --- | --- | --- | --- | --- | --- | --- | --- | --- | --- | --- | --- | --- | --- | --- | --- | --- | --- | --- | --- | --- | --- | --- | --- | --- | --- | --- | --- | --- | --- | --- | --- | --- | --- | --- | --- | --- | --- | --- | --- | --- | --- | --- | --- | --- | --- | --- | --- | --- | --- | --- | --- | --- | --- | --- | --- | --- | --- | --- | --- | --- | --- | --- | --- | --- | --- | --- | --- | --- | --- | --- | --- | --- | --- | --- | --- | --- | --- | --- | --- | --- | --- | --- | --- | --- | --- | --- | --- | --- | --- | --- | --- | --- | --- | --- | --- | --- | --- | --- | --- | --- | --- | --- | --- | --- | --- | --- | --- | --- | --- | --- | --- | --- | --- | --- | --- | --- | --- | --- | --- | --- | --- | --- | --- | --- | --- | --- | --- | --- | --- | --- | --- | --- | --- | --- | --- | --- | --- | --- | --- | --- |

| **Men’s questionnaire** | |
| --- | --- |
|  | |
| **man’s information panel** | |
| *This questionnaire is to be administered to all men in selected households who are age 15 through 54 who have a child under 5 in the household (indicated in HL9A AND HL10A)*  *A separate questionnaire should be used for each eligible man.* | |
| **MQ1**. Province _____________________________ | **MQ2**. County _____________________________ |
| **MQ3**. Cluster number: _____ _____ | **MQ4**. Household number: _____ _____ _____ |
| **MQ5**. Man’s name: | **MQ6**. Man’s line number: |
| Name | ___ ___ |
| **MQ7**. Interviewer’s name and number: | **MQ8**. Day / Month / Year of interview: |
| Name ___ ___ | ___ ___ /___ ___ / 2 0 1 ___ |
| \| *Repeat consent process if not already read to this man:*  READ THE CONSENT FORM (HARD COPY) TO THE RESPONDENT; TAKE CONSENT. ENSURE THAT THEY SIGN THE CONSENT FORM IF THEY AGREE TO PARTICIPATE”.  **If the man is 15-17 years old and married, read the consent script to any person the man is comfortable with and the assent script to the man. You MUST obtain the consent of the parent or guardian and assent of the man before proceeding** \| \| --- \| \| *If greeting at the beginning of the household questionnaire has already been read to this man, then read the following:*  Now I would like to talk to you more about your health and other topics. This interview will take about ***insert number*** minutes. Again, all the information we obtain will remain strictly confidential and anonymous. \| | |
| **MQ9**. Result of man’s interview | Completed 01  Not at home 02  Not competent 03  Deferred 04  Refused 05  Partially completed 06  Other (*specify*) 96 |

| 1. Have you ever attended formal education? | Yes 1  No. 2 | 2🡪M3 |
| --- | --- | --- |
| 1. What is the highest level of school you completed? | Preschool 0  Primary 1  Secondary 2  Higher 3 | 0**🡪**M4  3**🡪**M4 |
| 1. What is the highest grade you completed at that level?   *If the first grade at this level is not completed,  enter “00”.* | Grade __ __ |  |
| 1. How many years old is your **youngest** child?   If older than 1 year, record years | Less than 1 year 0  Age in years __ __ | IF OVER 5 🡪END SURVEY |
| 1. Does this child live in the household? | Yes 1  No. 2 | 2🡪END SURVEY |
| 1. Who usually makes decisions about making major household purchases? | Respondent 1  Wife/Partner 2  Respondent and Wife/Partner 3  Father or Father-in-law 4  Mother or Mother in-law 5  Other family members 6  Other (*specify*) 96 |  |
| 1. Who usually makes decisions about health care for yourself? | Respondent 1  Wife/Partner 2  Respondent and Wife/Partner 3  Father or Father-in-law 4  Mother or Mother in-law 5  Other family members 6  Other (*specify*) 96 |  |
| 1. Who usually makes decisions about health care for your wife/partner? | Respondent 1  Wife/Partner 2  Respondent and Wife/Partner 3  Father or Father-in-law 4  Mother or Mother in- law 5  Other family members 6  Other (*specify*) 96 |  |
| Now I would like to talk about family planning - the various ways or methods that a couple can use to delay or avoid a pregnancy. Have you ever heard of (METHOD)? | | |
| M8.1. Female Sterilization.  PROBE: Women can have an operation to avoid having any more children. | Yes 1  No 2 |  |
| M8.2. Male Sterilization.  PROBE: Men can have an operation to avoid having any more children. | Yes 1  No 2 |  |
| M8.3. IUD.  PROBE: Women can have a loop or coil placed inside them by a doctor or a nurse which can prevent pregnancy for one or more years. | Yes 1  No 2 |  |
| M8.4. Injectables.  PROBE: Women can have an injection by a health provider that stops them from becoming pregnant for one or more months. | Yes 1  No 2 |  |
| M8.5. Implants.  PROBE: Women can have one or more small rods placed in their upper arm by a doctor or nurse which can prevent pregnancy for one or more years. | Yes 1  No 2 |  |
| M8.6. Pill.  PROBE: Women can take a pill every day to avoid becoming pregnant. | Yes 1  No 2 |  |
| M8.7. Condom.  PROBE: Men can put a rubber sheath on their penis before sexual intercourse. | Yes 1  No 2 |  |
| M8.9. Female Condom.  PROBE: Women can place a sheath in their vagina before sexual intercourse. | Yes 1  No 2 |  |
| M8.10. Emergency Contraception.  PROBE: As an emergency measure, within three days after they have unprotected sexual intercourse, women can take special pills to  prevent pregnancy. | Yes 1  No 2 |  |
| M8.11. Standard Days Method.  PROBE: A woman uses a string of colored beads to know the days  she can get pregnant. On the days she can get pregnant, she uses a condom or does not have sexual intercourse. | Yes 1  No 2 |  |
| M8.12. Lactational Amenorrhea Method (LAM).  PROBE: Up to six months after childbirth, before the menstrual period  has returned, women use a method requiring frequent breastfeeding  day and night. | Yes 1  No 2 |  |
| M8.13. Rhythm Method.  PROBE: To avoid pregnancy, women do not have sexual intercourse  on the days of the month they think they can get pregnant. | Yes 1  No 2 |  |
| M8.14. Withdrawal.  PROBE: Men can be careful and pull out before climax. | Yes 1  No 2 |  |
| M8.15. In the last few months have you:   1. Heard about family planning on the radio? 2. Seen anything about family planning on the television? 3. Read about family planning in a newspaper or magazine? 4. Received a voice or text message about family planning on a mobile phone? 5. Posters/billboards 6. Friends and family members 7. Social media | Yes NO  radiO 1 2  Television 1 2  Newspaper 1 2  Mobile phone 1 2  Posters/billboards 1 2  Friends and family members 1 2  Social media 1 2 |  |
| 1. Are you or your partner currently doing something or using any method to delay or avoid getting pregnant? | Yes 1  No 2  Prefer not to answer 66  Don’t know 98 | 2🡪M11 |
| 1. What are you currently doing to delay or avoid a pregnancy?   Do not prompt.  If more than one method is mentioned, circle each one. | Female sterilization A  Male sterilization B  IUD C  Injectables D  Implants E  Pill F  Male condom G  Female condom H  Diaphragm I  Foam / Jelly J  Lactational amenorrhoea method (LAM) K  Periodic abstinence / Rhythm L  Withdrawal M  Standard days method N  Other (*specify*) X  Prefer not to answer Y |  |
| 1. Would you say that using contraception is mainly your decision, mainly your wife’s/partner’s decision or did you both decide together? | Mainly respondent 1  Mainly wife/partner 2  Joint decision 3  Other (*specify*) X |  |
| M12. NOW I WOULD LIKE TO ASK ABOUT your knowledge regarding age of marriage, REGARDLESS if you are CURRENTLY married or not or AT WHAT AGE YOU WERE MARRIED.  what are issues or CONSEQUENCES OF early marriage that you have heard of?  **DO NOT PROMPT.**  **CIRCLE ALL THAT ARE MENTIONED.**  **IF NONE ARE MENTIONED, SELECT 99.** | \| NONE-NO CONSEQUENCES/ISSUES \| 1 \| \| --- \| --- \| \| AGAINST THE LAW \| 2 \| \| DENIES BASIC RIGHT TO CHOOSE \| 3 \| \| RISK OF DEATH WITH EARLY PREGNANCY \| 4 \| \| MORE VULNERABLE TO STIs, INCL.HIV \| 5 \| \| MORE LIKELY TO DROP OUT OF SCHOOL \| 6 \| \| MORE VULNERABLE TO ABUSE \| 7 \| \| UNPLANNED PREGNANCY \| 8 \| \| RISK OF MENTAL ILLNESS \| 9 \| \| SOCIAL ISOLATION \| 10 \| \| ECONOMIC DEPENDENCE \| 11 \| \| WORSE NUTRITIONAL OUTCOMES \| 12 \| \| PREFER NOT TO ANSWER \| 66 \| \| DON’T KNOW \| 99 \| |  |
| M13. finally, I would like to ask about your knowledge on forms of violence. Can you name the various forms of VIOLENCE THAT A WOMAN OR FEMALE ADOLESCENT could EXPERIENCE?  **DO NOT PROMPT.** CIRClE ALL RESPONSES THAT ARE SAID.  AFTER, ASK ARE THERE ANY OTHER THINGS? | **PHYSICAL VIOLENCE**  BEATING/HITTING/KICKING 1  FEMALE GENITAL MUTILATION/INITIATION 2  BURNING 3  **SEXUAL VIOLENCE**  RAPE OR ATTEMPTED RAPE 4  SEXUAL SLAVERY 5  TRAFFICKING 6  SEXUAL HARASSMENT 7  SEXUAL ASSAULT 8  DENIAL OF RIGHT TO USE CONTRACEPTION 9  FORCED ABORTION 10  MOLESTATION 11  FORCED EARLY MARRIAGE 12  NOT DISCLOSING HIV STATUS 13  **EMOTIONAL VIOENCE**  PSYCHOLOGICAL ABUSE 14  THREATS 15  VERBAL ABUSE 16  STALKING 17  **FINANCIAL VIOLENCE**  WITHHOLDING MONEY 18  STEALING 19  PROHIBITING WORKING OR SCHOOL 20  DON’T KNOW 99  PREFER NOT TO ANSWER 66 |  |

| QUESTIONS FOR MAN WITH CHILD UNDER 2 QUESTIONNAIRE | | | | | |
| --- | --- | --- | --- | --- | --- |
| *These questions are to be administered ONLY to men with children under the age of two.*  Check M3  🞎 *Youngest child is age 0-2 years ⇨ Continue with DM1.*  🞎 *Youngest child is greater than 2 years ⇨ DM11* | | | | | |
| 1. When (name’s) mother was pregnant with (NAME) did she have any antenatal check-ups? | | Yes 1  No. 2  Prefer not to answer 66  Don’t know 98 | |  | |
| 1. Were you ever present during any of those antenatal care check-ups? | | Yes 1  No. 2  Don’t know 98 | |  | |
| **Now I would like to ask you some questions about danger signs during pregnancy, labour and newborn health** | | | | | |
| 1. Are you aware of any danger signs during pregnancy? | | Yes 1  No. 2 | | 2🡪DM5 | |
| 1. Could you please name any danger signs during pregnancy that you are aware of?   Probe: Any other danger signs?  Keep asking for more danger signs until the participant cannot recall any additional signs. Circle all that are mentioned, but do not prompt with any suggestions. | | Difficulty breathing A  Fatigue/tiredness B  Vaginal bleeding C  Baby not moving D  Loss of consciousness E  Convulsions F  Headache and blurred vision G  Early labour pain before term H  Vaginal discharge I  Signs of malaria  (feeling cold, fever, vomiting) J  High blood pressure K  Swelling of face and arms L  Does not know any of the above M  Other (*specify*) X | |  | |

| 1. Are you aware of any danger signs during labour and childbirth? | Yes 1  No. 2 | 2**🡪DM7** |
| --- | --- | --- |
| 1. Could you please name any danger signs during labour and childrbirth?   Probe: Any other danger signs?  *Keep asking for more danger signs until the participant cannot recall any additional signs. Circle all that are mentioned, but do not prompt with any suggestions.* | Severe vaginal bleeding (> 12 hours) A  Prolonged labour B  Convulsions C  Retained placenta D  Baby lying sideways or upsidedown E  Fever F  Green or brown waters G  Strong headache H  Blurred or double vision I  Sudden, steady severe pain at the top of the belly J  Overactive reflexes K  High blood pressure L  Protein in the urine M  Does not know any of the above N  Other (*specify*) X |  |

| 1. Are you aware of any danger signs after delivery? | | Yes 1  No. 2 | 2**🡪DM9** |  |
| --- | --- | --- | --- | --- |
| 1. What are some of the danger signs that you should watch out for after delivery?   Probe: Any other danger signs?  Keep asking for more danger signs until the participant cannot recall any additional signs. Circle all that are mentioned, but do not prompt with any suggestions.  **DO NOT PROMPT**  **MULTIPLE RESPONSES** | | High Fever, lower abdominal pain or foul smelling discharge (infection) A  Severe headache, blurred vision, high blood pressure B  Convulsions or fits (eclampsia) C  Heavy vaginal bleeding (PPH) D  Urinary or fecal incontinence (obstetric fistula) E  Extreme tiredness, Anemia F  Anxiety or depression (puerperal psychosis) G  Breast problems (engorgement, sore, cracked bleeding or inverted nipples) H  Does not know any of the above I  Other (*specify*) X |  |  |
| 1. Are you aware of any danger signs for newborns? | | Yes 1  No. 2 | | 2🡪DM11 |
| 1. Could you please name any danger signs for newborns?   Probe: Any other danger signs?  Keep asking for more danger signs until the participant cannot recall any additional signs. Circle all that are mentioned, but do not prompt with any suggestions. | | Convulsions/spasms/rigidity A  Movement only when stimulated or no movement,  even when stimulated B  Not feeding well C  Fever D  Difficult/fast breathing E  Lethargy/unconsciousness F  Does not know any of the above G  Other (*specify*) X | |  |

| *These questions are to be administered to ALL men with children under the age of five.*  *Check M3: Proceed to DM11 if child is reported under the age of 5*  *If no child under 5, end survey for this man* | | |
| --- | --- | --- |
| 1. Sometimes children have severe illness and should be taken to a health facility. What types of symptoms would cause you to take a child under the age of 5 to a health facility right away?   Probe: Any other symptoms?  *Keep asking for more symptoms until the participant cannot recall any additional symptoms. Circle all symptoms mentioned, but do not prompt with any suggestions.* | Child not able to drink or breastfeed A  Child becomes sicker B  Child develops a fever C  Child has fast breathing D  Child has difficulty breathing E  Child has blood in stool F  Child is drinking poorly G  Child is vomiting H  Child has convulsions I  Child is lethargic J  Child is unconscious K  Child doesn’t cry L  Child’s skin is yellow M  Other (specify) X |  |
| 1. When a child has diarrhea, how much should he or she be given to drink; more than usual, the same as usual, less than usual, or should he or she not be given anything to drink at all? | More than usual 1  About the same 2  Less than usual 3  Nothing to drink 4  Don’t know 98 |  |
