# Supplementary material for: Factors associated with use of long-acting reversible and permanent contraceptives among married women in rural Kenya: A community-based cross-sectional study in Kisii and Kilifi counties
Source: PLoS One. 2022 Oct 6;17(10):e0275575. doi: 10.1371/journal.pone.0275575 (PMC9536593; doi:10.1371/journal.pone.0275575)
Supplement: Attachment — Submitted filename: PONE-D-21-23920_Comments.docx [file pone.0275575.s003.docx]

**PONE-D-21-23920**

Long-acting and permanent contraceptives use and associated factors among married women in rural Kenya: a community-based cross-sectional study

Thanks for the opportunity to review this manuscript. The paper discusses a very relevant topic in low- and middle-income countries (LMICs) where contraceptive use, especially use of LAPM remains poor despite many years of investment on family planning programmes. While millions of women across LMICs would like to space or limit their number of children, non-use of contraceptives remains high among them despite their sexual exposure and an expressed intention to avoid pregnancy. The authors use endline data from a health programme to assess prevalence and factors associated with the use of LAPM among married women in rural Kenya. I have some observations which if addressed would improve the paper.

**TITLE:**

LN 1-3: ‘Long-acting and permanent contraceptives use and associated factors among married women in rural Kenya: a community-based cross-sectional study’ Consider changing the title to ‘ *Factors associated with use of long-acting and permanent methods among married women in rural Kenya: a community-based cross-sectional study’*

**ABSTRACT:**

Include the sample size

**BACKGROUND:**

Generally, the background of this paper should be strengthened to make clear the new knowledge gap the study is filling. There are numerous studies on factors associated with use of LAPM including some from sub-Saharan Africa which authors should consider highlighting.

LN 46: Authors wrote ‘Shorter birth intervals are characterized by maternal, foetal, and

47 infant health problems’…There need to link this sentence with the first so that it is not left hanging. Also, consider replacing ‘health problems’ with’ negative health outcomes

LN 49: I would recommend to the authors to define what CPR means for readers not well versed in the field

LN 54: indicate the mandate of NCPD when it comes to FP?

**METHODS:**

The study is based on endline data from a program on improving Access to Quality Care and Extending and Strengthening Health Systems in Kilifi and Kisii counties of Kenya. Given that this programme also promoted FP, the results could be biased unless the analysis controlled for participation. There is need to describe efforts to address this particular bias. Alternatively, authors should consider using baseline data instead. Is it possible to indicate the number of participants who adopted a method including LAPM between baseline and endline?

Include a description of the study design under the method’s section. If cross-sectional, was it a repeated cross-sectional design?

The outcome variable ‘use of LAPM’ is dichotomous so logistic regression would be preferable.

It is not clear why the authors included ‘Heard about FP on social media’ as one of the independent variables. It appears superfluous as it does not have any influence in using LAPM relative to non-LAMP. It is so missing from the list of IV listed in LN140-150

**RESULT:**

Table 1- column 2, the percentage for each variable should add up to 100%

Ln 193: Authors wrote ‘The prevalence of LAPM use among women using contraceptives….’ Should read ‘The prevalence of LAPM use among married women using contraceptives… The result should be limited to only married women.

LN 194: Use of implants (24.5%) and female sterilization (7.1%) is more than double what was reported in the last KDHS. We need to see more in the discussion on what could be contributing to such a high increase in the study settings

LN 202: Table 2. It is interesting that some of the variables are insignificant on bivariate model but significant on the multivariate model. For example, age, future fertility preference and CEB. This is problematic and makes interpretation open to doubt. There could be confounding and collinearity issues that authors should address.

**DISCUSSION:**

LN 220-222, Authors wrote ‘*The findings could also be attributed to the possible indirect effects of the AQCESS project through community activities that encouraged FP utilization’* As already mentioned above, the intervention could bias the findings, therefore there need to control for participation. What elements of FP did the AQCESS project promote Did it only focus on LAPM or other methods as well? If both, then we expect an increase in the use of both LAPM and non-LAPM methods.

LN 238-243: In addition to demand- and supply-side barriers mentioned, there is also provider-related bias which may influence contraceptive uptake. Partner/Husband’s approval of the methods also play a big role especially in patriarchal societies where men dominate everything including reproductive health decisions.

LN 245: Authors wrote ‘ The higher odds of LAPM use among younger women may be attributed to their need to avoid unintended pregnancies and optimize birth spacing compared to older women. I find it difficult to accept this given that on the bivariate model, the association between age and use of LAPM is not significant. Generally, we expect use of LAPM to increase by age.

**OVERALL COMMENT:**

The article should be checked for grammatical errors throughout
