# Supplementary material for: Factors associated with use of long-acting reversible and permanent contraceptives among married women in rural Kenya: A community-based cross-sectional study in Kisii and Kilifi counties
Source: PLoS One. 2022 Oct 6;17(10):e0275575. doi: 10.1371/journal.pone.0275575 (PMC9536593; doi:10.1371/journal.pone.0275575)
Supplement: Attachment — Submitted filename: Responses to Reviewers Comments.docx [file pone.0275575.s004.docx]

| **Reviewers’ Comments** | **Responses** |
| --- | --- |
| **Reviewer 1** |  |
| 1. The article addresses an important topic on the use of long-acting and permanent contraceptives among married women in rural Kenya and the associated factors. The topic is important in Kenya for several reasons.  - The use of effective contraceptives is important for women to prevent unintended pregnancies that might pose serious health and social complications such as unsafe abortions and maternal/child morbidity and mortality. Women will be able to attain more in education and economic status if they can get children when they want. For the country, to reduce high costs incurred in maternal/child health care, unwanted fertility as well as the high population growth rate. - Increased use of these contraception which are more effective will address the challenge of unmet need for contraception in Kenya which is high 22%. Kenya is currently putting interventions towards achieving zero unmet need for contraception in line with its ICPD25 country commitments made in 2019 at the Nairobi Summit of the International Conference on Population and Development 25th Anniversary. - More use of these contraceptive methods will help reduce contraceptive discontinuation which is high at 31% and waters down the high contraceptive prevalence rate the country has attained. - It addresses the issue of contraception among the poor and marginalized setting: This is important because it is rural and hence the need for more exposure to contraceptive information and services for the women. |  |
| 2. Methodology  - Data- The data is original, collection methods and quality controls reported are important for credibility. Quality assurance is given - Model – The GEE regression model is appropriate for the population based survey which was using clustered data - Sampling – The sampling gives focus to inclusivity to avoid bias. Numbers of women interviewed, and the sampling procedures are adequate - Exclusion criteria reported is important. Fig 1 shows the issues. - Results- They are well presented in statistical language and discussed but improvements can be done on the presented information in the tables - References- They are recent, relevant and therefore appropriate - Recommendation – The paper is of publishable quality but needs some revisions and English language editing. |  |
|  |  |
| 3. Lines 1-3 Title – add Kisii and Kilifi at the end – This will create more interest in the study   “*Long-acting and permanent contraceptives use and associated factors among married women in rural Kenya: a community-based cross-sectional study in Kisii and Kilifi Counties*” | Thanks for the suggestion. We have revised the title to read “Factors associated with the use of long-acting reversible and permanent contraceptives among married women in rural Kenya: a community-based cross-sectional study in Kisii and Kilifi counties” |
| 4. Lines 30 Use regression model instead of approach – equations regression model…. | Revised appropriately |
| 5. Lines 41 Insert the word rural after targeting – LAPM targeting rural women | Revised appropriately |
| 6. Lines 45 Rewrite the sentence for clarity – Contraception helps to space and limit the number of children and thus prevents unintended pregnancies and unsafe abortions | The paragraph has been revised to read better  Revised appropriately |
| 7. Lines 46 Add sometimes because it is not always the case that health problems arise -intervals are sometimes characterized…. | Revised appropriately |
| 8. Lines 53-57 Rewrite the sentence to shorten it for clarity | Revised appropriately. The paragraph now reads: “” |
| 9. Line 56 –add mCPR after CPR | Revised appropriately |
| 10. Line 56 – change contraceptive to contraception--modern CPR (mCPR)…. - any method of contraception….. | Revised appropriately |
| 11. Lines 60/61 Improve clarity – long-acting reversible contraception (LARC) or permanent methods | Revised appropriately |
| 12. Lines 64 Add Citation besides [15] – I propose add citation of KDHS 2014 which presents discontinuation data | The reference has been added |
| 13. Lines 66-68 Can add a sentence on what the Government direction is to back up the information-Refer to the National Family Planning Costed Implementation Plan, 2017-2020 | The section has been revised |
| 14. Lines 83 Addition to sentence – rural Kenya based on a community study in Kisii and Kilifi Counties. | Revised appropriately |
| 15. Lines 87-88 Sentence improvement – The counties may not be called rural, but the sub counties can be rural – February 2020 in four rural sub-counties of Kisii and Kilifi Counties | Revised appropriately |
| 16. Lines 97 Correction on the teen pregnancy rates – Kisii is 18.4 and Kilifi 21.8 – Citation [23] – 18-22% | Revised appropriately |
| 17. Add national average of 18% – Reference (KDHS,2014) | Revised appropriately |
| 18. Lines 109-112 The sentence needs rewriting for more clarity – Especially add something on the issue of 80% power and 20% increase, design effect of two | Revised appropriately |
| 19. Lines 181-183 Mention age of youngest and oldest also | Thanks, the study included women of reproductive age 15-49 years. This has been added in the text as well |
| 20. Lines 189 Table 1 – Change word characteristic to Variable | Revised appropriately |
| 21. Sample characteristic to Number/% | Revised appropriately |
| 22. Lines 201 Figure 2 - Change word current to currently | Revised appropriately |
| 23. Lines 202-203 Table 2 | Revised appropriately |
| 24. The bivariate and multivariate presentation can be improved to avoid congestion which is not friendly to a reader Change word characteristic to Variable | We have separated the table into Table 1 (respondents characteristics) and Table 2 (results of bivariable and multivariable logistic regression) for better readability |
| 25. Word multivariate may be better than multivariable | We have retained the use of ‘multivariable’ because we believed it is the correct terminologies as argued in the following articles: <https://doi.org/10.1016/j.jagp.2018.11.008> or <https://doi.org/10.1093/ntr/ntaa055> |
| 26. Maybe subdivide each into two: cOR 95% CI and aOR 95% CI | Revised appropriately |
| 27. Lines 207 Multivariable analysis – Word multivariate may sound better than multivariable | We have retained the use of ‘multivariable’ because we believed it is the correct terminologies as argued in the following articles: <https://doi.org/10.1016/j.jagp.2018.11.008> or <https://doi.org/10.1093/ntr/ntaa055> |
| 28. Lines 208 Rewriting – The odds of using LAPM were …… | Revised appropriately |
| 29. Lines 213 Tense – have ever – Women who had given birth….. | Revised appropriately |
| 30. Lines 214 Rewriting/tense odds of using LAPM compared to those who had… | Revised appropriately |
| 31. Lines 217 Rewriting – add found in-lower than 57% found in a community FP | Revised appropriately |
| 32. Lines 220 Rewriting – add modern – uptake of modern contraceptive services | Revised appropriately |
| 33. Lines 223 Rewriting – add organizations-non-governmental organizations targeted… | Revised appropriately |
| 34. Lines 224 Make plural – such as low numbers of trained providers | Revised appropriately |
| 35. Lines 228 Cut sentence for clarity – at a low cost [36]. They may be | Revised appropriately |
| 36. Lines 230 Improve clarity – And in conformity to previous studies | Revised appropriately |
| 37. Lines 232 Improve clarity – remove when needed- easily removed without…... | Revised appropriately |
| 38. Lines 233 Improve clarity – implants more than IUCDs [20, 40]. | Revised appropriately |
| 39. 235 Remove our – in the study setting | Revised appropriately |
| 40. Lines 240 Improve clarity – about the use of permanent methods because they are irreversible and provider hesitancy to remove LARCs [34]. | Revised appropriately |
| 41. Lines 241-243 Rewrite sentence for clarity | Revised appropriately |
| 42. Lines 245 Improve clarity – higher odds of using LAPM among | Revised appropriately |
| 43. Lines 247 Improve clarity – explain their higher use of implants | Revised appropriately |
| 44. Lines 248-250 Improve clarity – The study also found that women in the richest wealth tercile were more likely to use LAPM than women in the poorest wealth tercile, which mirrors findings in other studies [44, 45]. | Revised appropriately |
| 45. Lines 255/256 Improve clarity – settings were rural with poor geographical accessibility to health facilities and with limited human healthcare resources | Revised appropriately |
| 46. Lines 257 Add more – with 3–5 children were more likely to use LAPM | Revised appropriately |
| 47. Lines 258-260 Rewrite sentence for clarity – LAPM offers efficient, long-term protection against pregnancy, and that these women may have achieved their desired number of children or want to space their births. | Revised appropriately |
| 48. Lines 260-261 Average children is 3.9 (KDHS,2014) – Improve clarity - Besides, Kenyan women have on average 3.9 children ever born [11], which is consistent with the study findings. | Revised appropriately |
| 49. Lines 269 Improve clarity – only possible associations | Revised appropriately |
| 50. Lines 274 Improve clarity – omit and-ever tercile, those who did not want more children and those had given birth to 3–5 children. | Revised appropriately |
| 51. Lines 276-277 Rewrite sentence for clarity – LAPM among women in rural areas and low socioeconomic status. Moreover, further research is needed to investigate the effects of other factors which…... | Revised appropriately |
| 52. References  - Lines 327 KDHS year of publication – 2015 - Lines 342 Ref 16 – i) Check authors names are written correctly – ii) DHS Analytical Studies 20 - Lines 362 Ref 21 – Complete Report No | Revised appropriately |
| **Reviewer 2** |  |
| 1. Thanks for the opportunity to review this manuscript. The paper discusses a very relevant topic in low- and middle-income countries (LMICs) where contraceptive use, especially use of LAPM remains poor despite many years of investment on family planning programmes. While millions of women across LMICs would like to space or limit their number of children, non-use of contraceptives remains high among them despite their sexual exposure and an expressed intention to avoid pregnancy. The authors use endline data from a health programme to assess prevalence and factors associated with the use of LAPM among married women in rural Kenya. I have some observations which if addressed would improve the paper. |  |
| 2. **Title**: LN 1-3: ‘Long-acting and permanent contraceptives use and associated factors among married women in rural Kenya: a community-based cross-sectional study’ Consider changing the title to ‘*Factors associated with use of long-acting and permanent methods among married women in rural Kenya: a community-based cross-sectional study*’ | Thanks for the suggestion. We have revised the title to read “*Factors associated with the use of long-acting reversible and permanent contraceptives among married women in rural Kenya: a community-based cross-sectional study in Kisii and Kilifi counties*” |
| 3. **Abstract**: Include the sample size | Thank you for the comment. We have revised the abstract and included the sample size. |
| ***Background*** |  |
| 4. Generally, the background of this paper should be strengthened to make clear the new knowledge gap the study is filling. There are numerous studies on factors associated with use of LAPM including some from sub-Saharan Africa which authors should consider highlighting. | Thank you for the comment. We have revised the background of the study to better reflect the knowledge gap the study is filling. |
| 5. LN 46: Authors wrote ‘Shorter birth intervals are characterized by maternal, foetal, and 47 infant health problems’…There need to link this sentence with the first so that it is not left hanging. Also, consider replacing ‘health problems’ with’ negative health outcomes | Thanks you for the comment. A linking sentence has been added in the text |
| 6. LN 49: I would recommend to the authors to define what CPR means for readers not well versed in the field | Thank you for the comment. We have defined CPR |
| 7. LN 54: indicate the mandate of NCPD when it comes to FP? | Thank you for the comment. We have now included the mandate of NCPD |
| ***Methods*** |  |
| 8. The study is based on endline data from a program on improving Access to Quality Care and Extending and Strengthening Health Systems in Kilifi and Kisii counties of Kenya. Given that this programme also promoted FP, the results could be biased unless the analysis controlled for participation. There is need to describe efforts to address this particular bias. Alternatively, authors should consider using baseline data instead. Is it possible to indicate the number of participants who adopted a method including LAPM between baseline and endline? | Thank you for the comment. We agree that use of both the baseline and end-line would have been appropriate. Family planning was not one of the key interventions of the project. Community sensitization on family planning was the only activity included as part of the project, no FP product was given out to the women as part of the project.  We agree controlling for participation would have given the study more information. However, this is a repeat cross-sectional study where there were cases of new participants included in the end line that actually did not participate in the baseline.  We have added the prevalence of contraceptive use for each method for baseline and end line for review. |
| 9. Include a description of the study design under the method’s section. If cross-sectional, was it a repeated cross-sectional design? | Revised appropriately to include the study design as repeat cross-section design |
| 10. The outcome variable ‘use of LAPM’ is dichotomous so logistic regression would be preferable. | We have reanalysed the data using logistic regression. This was arrived at since the women were interviewed only once during the end line survey (cross-sectional) resulting to independent observation per woman |
| 11. It is not clear why the authors included ‘Heard about FP on social media’ as one of the independent variables. It appears superfluous as it does not have any influence in using LAPM relative to non-LAMP. It is so missing from the list of IV listed in LN 140-150 | Thank you for the comment. This was a mistake from us. We have corrected it to “Media exposure to FP messages” which was a composite of radio, TV, and social media. We have also referenced it appropriately as it has been shown to be associated with family planning use. |
| ***Results*** |  |
| 12. Table 1 – column 2, the percentage for each variable should add up to 100% | Thank you for the comment. This was a mistake from us. We have corrected the Table in the two newly created Table 1. |
| 13. Ln 193: Authors wrote ‘The prevalence of LAPM use among women using contraceptives….’ Should read ‘The prevalence of LAPM use among married women using contraceptives… The result should be limited to only married women. | Thank you for the comment. We have revised it appropriately |
| 14. LN 194: Use of implants (24.5%) and female sterilization (7.1%) is more than double what was reported in the last KDHS. We need to see more in the discussion on what could be contributing to such a high increase in the study settings | Thanks for your comment, we have added a comment that we thought could be attributed to the increase.  The increase in use could be attributed to the efforts of the CHVs on sensitizing the community on the benefits of family planning methods which includes LAPMs |
| 15. MLN 202: Table 2. It is interesting that some of the variables are insignificant on bivariate model but significant on the multivariate model. For example, age, future fertility preference and CEB. This is problematic and makes interpretation open to doubt. There could be confounding and collinearity issues that authors should address. | Thanks for your comments, we have changed the model from GEE to binary logistic regression. All variables that are significant in the bivariate are the same ones significant in the multivariable analysis. We have checked the presence of collinearity in the process of model building and found none of the variables were related with each other. |
| ***Discussion*** |  |
| 16. LN 220-222, Authors wrote ‘The findings could also be attributed to the possible indirect effects of the AQCESS project through community activities that encouraged FP utilization’ As already mentioned above, the intervention could bias the findings, therefore there need to control for participation. What elements of FP did the AQCESS project promote Did it only focus on LAPM or other methods as well? If both, then we expect an increase in the use of both LAPM and non-LAPM methods. | There was no intervention targeted to FP apart from community sensitization through CHVS. There was also no FP product given out as part of the projects as the women interested in using FP were referred to their nearest health facilities to get the product. The survey was a repeat cross-sectional design and most of the participants who participated in the baseline were not included in the end line.  Our analysis of the prevalence of different FP is attached for your review and it shows an increase in all the methods between baseline and end line |
| 17. LN 238-243: In addition to demand- and supply-side barriers mentioned, there is also provider-related bias which may influence contraceptive uptake. Partner/Husband’s approval of the methods also play a big role especially in patriarchal societies where men dominate everything including reproductive health decisions. | We agree with this statement and thanks, this is one of the variables that the project should have measured but was left out. We have included this as a limitation of our study |
| 18. LN 245: Authors wrote ‘The higher odds of LAPM use among younger women may be attributed to their need to avoid unintended pregnancies and optimize birth spacing compared to older women. I find it difficult to accept this given that on the bivariate model, the association between age and use of LAPM is not significant. Generally, we expect use of LAPM to increase by age. | We have noted this and changed analysis in line with the study design, the interpretation of the results have been described appropriately in line with the results. Our analysis still shows odds of using LAPM decreases with age, though not significant finding. However, in Table 2 the use increase with age up to age 30-39 years and declines among the 40-49 age-group |
| 19. The article should be checked for grammatical errors throughout | Thanks, we have received the whole article and checked for grammar |
|  |  |
| **Editor’s Comments/Journal Requirements** |  |
| 1. Please ensure that your manuscript meets PLOS ONE's style requirements, including those for file naming. | Revised appropriately |
| 2. Please include additional information regarding the survey or questionnaire used in the study and ensure that you have provided sufficient details that others could replicate the analyses. For instance, if you developed a questionnaire as part of this study and it is not under a copyright more restrictive than CC-BY, please include a copy, in both the original language and English, as Supporting Information. | The questionnaire adapted questions from the demographic and health surveys and also from multiple indicator clusters surveys. These surveys have validated question since they have been used to collect data previously in the different national surveys. We have added these statement in the text under data collection tool and procedure.  A copy in English version is attached as supporting information. |
| 3. Thank you for stating the following financial disclosure: "The funders had no role in analysis, decision to publish, or preparation of the manuscript"   At this time, please address the following queries:   a) Please clarify the sources of funding (financial or material support) for your study. List the grants or organizations that supported your study, including funding received from your institution. b) State what role the funders took in the study. If the funders had no role in your study, please state: “The funders had no role in study design, data collection and analysis, decision to publish, or preparation of the manuscript.” c) If any authors received a salary from any of your funders, please state which authors and which funders. d) If you did not receive any funding for this study, please state: “The authors received no specific funding for this work.”   Please include your amended statements within your cover letter; we will change the online submission form on your behalf. | The cover letter amended appropriately. Kindly adjust |
| 4. Thank you for stating the following in the Acknowledgments Section of your manuscript:   "We are grateful to the Government of Canada, and the Aga Khan Foundation Canada for funding the end line survey from which data were extracted for this study. We acknowledge the Ministry of Health, and the Kisii and Kilifi County governments, and the village elders, or support in conducting this study. We are grateful to participants who consented to the interviews and the AQCESS teams in Kenya (Kennedy Mulama, Rachel Odhiambo, Michaela Mantel, and Lucy Nyaga)."  We note that you have provided funding information that is not currently declared in your Funding Statement. However, funding information should not appear in the Acknowledgments section or other areas of your manuscript. We will only publish funding information present in the Funding Statement section of the online submission form.  Please remove any funding-related text from the manuscript and let us know how you would like to update your Funding Statement. Currently, your Funding Statement reads as follows:  "The funders had no role in analysis, decision to publish, or preparation of the manuscript"  Please include your amended statements within your cover letter; we will change the online submission form on your behalf. | Funding statement removed, thanks. |
| 5. We note that you have indicated that data from this study are available upon request. PLOS only allows data to be available upon request if there are legal or ethical restrictions on sharing data publicly. For more information on unacceptable data access restrictions, please see <http://journals.plos.org/plosone/s/data-availability#loc-unacceptable-data-access-restrictions>.   In your revised cover letter, please address the following prompts:   a) If there are ethical or legal restrictions on sharing a de-identified data set, please explain them in detail (e.g., data contain potentially sensitive information, data are owned by a third-party organization, etc.) and who has imposed them (e.g., an ethics committee). Please also provide contact information for a data access committee, ethics committee, or other institutional body to which data requests may be sent. b) If there are no restrictions, please upload the minimal anonymized data set necessary to replicate your study findings as either Supporting Information files or to a stable, public repository and provide us with the relevant URLs, DOIs, or accession numbers. For a list of acceptable repositories, please see <http://journals.plos.org/plosone/s/data-availability#loc-recommended-repositories>. c) We will update your Data Availability statement on your behalf to reflect the information you provide. | Anonymized dataset attached as S2 |
| 6. Please include captions for your Supporting Information files at the end of your manuscript, and update any in-text citations to match accordingly. Please see our Supporting Information guidelines for more information: <http://journals.plos.org/plosone/s/supporting-information> | Revise appropriately |
