# Supplementary material for: Factors associated with use of long-acting reversible and permanent contraceptives among married women in rural Kenya: A community-based cross-sectional study in Kisii and Kilifi counties
Source: PLoS One. 2022 Oct 6;17(10):e0275575. doi: 10.1371/journal.pone.0275575 (PMC9536593; doi:10.1371/journal.pone.0275575)
Supplement: Attachment — Submitted filename: comments to PONE-D-21-23920R1.docx [file pone.0275575.s005.docx]

Thank you for the opportunity to review this manuscript for the second time. While the authors addressed some issues raised in the first draft, some still need attention, particularly on the methodology. See below my main comments:

**General comments:**

The articles have improved but need to be reviewed further for language and grammatical mistakes.

**Introduction:**

Line 46: What goals are you referring to here? Please indicate because the goals could be many.

Line 47: Insert the article 'The' before World and REPLACE 'World health organization' with 'World Health Organization. Also, add the acronym in the bracket, i.e., (WHO)

Line: 51-56: Authors wrote that 'Globally, regions with low fertility rates tend to have a high contraceptive prevalence rate (CPR) compared to sub-Saharan Africa (SSA), where the CPR is still low (5)'. Please add the CPR prevalence to show the magnitude of the variation you refer to. Compare CPR in the following order, global, regional, and national.

Line 68: indicate why we have a high discontinuation rate for injectables and Pill. I think discontinuation rates are high for the hormonal methods including implants, due to health-related reasons, including side effects and erroneous perception that some methods cause infertility.

Line 73: indicate which methods constitute the LAPM methods (i.e., Intra-Uterine Devices (IUDs), Implants, Tubal ligation, and Vasectomy) and why they have become a strategy for reducing maternal morbidity and mortality.

Methods:

Line 105-117: This paragraph highlights the context of the study, but it is heavy on MNCH indicators. Consider adding FP indicators that are already available from 2014 KDHS.

Measurement:

The study has some methodological biases that should be addressed. I still believe this study would add value by comparing the baseline and endline data sets, even though the design was not longitudinal. As already pointed out in the first draft, using endline data without controlling for women's participation in the project creates biases in the result since the AQCESS project promoted the use of family planning through community sensitization by the community health volunteers (CHVs). The authors assertion that family planning was not one of the critical interventions of the project is wrong since CHV sensitized women to FP. There is a possibility that some of the women were reached with FP messages; others may have not. In line 226, the authors allude to the effects of the AQCESS project on improving uptake of LAPM, but the analysis does not inform that.

While it is okay to exclude from the analysis menopausal women as well as those who had a hysterectomy, it is not clear why the analysis excluded non-users and pregnant women. Remember, non-use is also a choice women make regarding contraceptive use. One way of addressing this is to group the outcome variable into three categories: using long-acting and permanent contraceptive methods (IUD, female sterilization, and implant), using other methods (short-acting and traditional), and not using any method, and applying a multinomial logistic regression model instead of binary logistic.

In the flow diagram showing the analysis sample, it appears the study collected data on the timing the respondent started using LAPM. The timing a woman started using LAPM should be controlled for in the analysis. Such a variable could also assuage some of the weaknesses highlighted above.

Line 169: specify the confounders controlled for in the analysis

Line 166-173: Did the analysis control for the clustering effects? It is very important because of the survey design used.

Line 229-232: Authors argue that some supply-side factors are barriers to LAPM use, but I did not see that in the analysis result.
