# Supplementary material for: Factors associated with use of long-acting reversible and permanent contraceptives among married women in rural Kenya: A community-based cross-sectional study in Kisii and Kilifi counties
Source: PLoS One. 2022 Oct 6;17(10):e0275575. doi: 10.1371/journal.pone.0275575 (PMC9536593; doi:10.1371/journal.pone.0275575)
Supplement: Attachment — Submitted filename: Reviewer.docx [file pone.0275575.s006.docx]

Dear Reviewer #2;

Thanks for taking time to review our article. The feedback given has really improved the write-up of this article. See below how we have addressed each of your comments.

Reviewer #2: Comments to the authors
Thank you for the opportunity to review this manuscript for the second time. While the authors addressed some issues raised in the first draft, some still need attention, particularly on the methodology. See below my main comments:

General comments:
The articles have improved but need to be reviewed further for language and grammatical mistakes.

Thanks, this is noted and revised accordingly.

Introduction:
Line 46: What goals are you referring to here? Please indicate because the goals could be many.

Development goals universal education and gender equality in career plans. The statement added in the background section lines 49
Line 47: Insert the article 'The' before World and REPLACE 'World health organization' with 'World Health Organization. Also, add the acronym in the bracket, i.e., (WHO)

Thanks, this is added in the document in line 50
Line: 51-56: Authors wrote that 'Globally, regions with low fertility rates tend to have a high contraceptive prevalence rate (CPR) compared to sub-Saharan Africa (SSA), where the CPR is still low (5)'. Please add the CPR prevalence to show the magnitude of the variation you refer to. Compare CPR in the following order, global, regional, and national.

A paragraph is added with these statistics under background section lines 58-64 with appropriate citations

Line 68: indicate why we have a high discontinuation rate for injectables and Pill. I think discontinuation rates are high for the hormonal methods including implants, due to health-related reasons, including side effects and erroneous perception that some methods cause infertility.

The high discontinuation rate of short-acting methods could be due to switching to more effective methods, contraceptive method stock-outs , prefer to conceive or fear of side effects associated with a particular method.

Thanks, we have added the following statement in lines 78-80

Line 73: indicate which methods constitute the LAPM methods (i.e., Intra-Uterine Devices (IUDs), Implants, Tubal ligation, and Vasectomy) and why they have become a strategy for reducing maternal morbidity and mortality.

Thanks, we have added examples in many instances within the document

Methods:
Line 105-117: This paragraph highlights the context of the study, but it is heavy on MNCH indicators. Consider adding FP indicators that are already available from 2014 KDHS.

Thanks, The FP indicators are available either at county levels or at a region levels. We did not get any literature specific to the areas studied in the two counties. We have included the CPR at the two counties in lines 121-122 from KDHS 2014.

Measurement:
The study has some methodological biases that should be addressed. I still believe this study would add value by comparing the baseline and endline data sets, even though the design was not longitudinal. As already pointed out in the first draft, using endline data without controlling for women's participation in the project creates biases in the result since the AQCESS project promoted the use of family planning through community sensitization by the community health volunteers (CHVs). The authors assertion that family planning was not one of the critical interventions of the project is wrong since CHV sensitized women to FP. There is a possibility that some of the women were reached with FP messages; others may have not. In line 226, the authors allude to the effects of the AQCESS project on improving uptake of LAPM, but the analysis does not inform that.

Thanks, we have included the baseline as well as endline data. It is however, worth nothing that the two populations are quite independent as not everyone who participated in the baseline was part of the end line survey. The study also did not asked the women who participated in the end line if they were part of the baseline. Due to this, we did not have a clear understanding on how this could be controlled in the analysis.

On the sensitization of the FP messages, the CHVs used a same curriculum with targeted messages for the households which included use of FP. As a result we assumed all the households received same messages during the project implementation. As indicated this was a project on maternal and newborn health with emphasis given to ANC, delivery and postpartum services.

Thanks, we agree that the analysis did not included the effect of the AQCESS project. However, among other unmeasured factors that could help in promoting access the uptake of LAPM, efforts of the CHVs under AQCESS project could not be ignored no matter how small it could have been. The project effort cannot directly be associated with the change since this was not measured. We have added this one of the limitation of this study.

While it is okay to exclude from the analysis menopausal women as well as those who had a hysterectomy, it is not clear why the analysis excluded non-users and pregnant women. Remember, non-use is also a choice women make regarding contraceptive use. One way of addressing this is to group the outcome variable into three categories: using long-acting and permanent contraceptive methods (IUD, female sterilization, and implant), using other methods (short-acting and traditional), and not using any method, and applying a multinomial logistic regression model instead of binary logistic.

Thanks, the study outcome was generated based on the question on current use of any method of contraceptive to delay or avoid pregnancy. Pregnant woman are already carrying a pregnancy and definitely was not on any method of contraceptive at the time of the study and were therefore not asked this question.

We have retained the outcome variables as binary in lines 158-164 to include using long-acting and permanent contraceptive methods (IUD, female sterilization, and implant), or not using that includes using short-acting and traditional and not using any method.

Thanks for your suggestion to use multinomial logistic regression instead of the binary logistic regression. Using this multinomial logistic model will change our study objective and from studying the use of LAPM to studying contraceptive use. Our study objective is to study the use of LAPM among married women of reproductive age. To motivate our argument further, multinomial regression is equivalent to fitting two binary logistic regression models simultaneously setting one of the groups as baseline category; that would have been (i) use of LAPM versus non-users of any methods of contraceptive and (ii) use of short-acting/traditional method versus non-users of any method of contraceptive. The first model (i) will leave out the short-acting group while the (ii) leaves out the LAPM group options which are not in line with our study objective. More details on multinomial logistic regression can be found in categorical data analysis second edition by Alan Agresti. PP 267-268 and categorical data analysis using the SAS system by Maura E.stokes, Charles S. Davis, and Gary G. Koch pp 257-259

In the flow diagram showing the analysis sample, it appears the study collected data on the timing the respondent started using LAPM. The timing a woman started using LAPM should be controlled for in the analysis. Such a variable could also assuage some of the weaknesses highlighted above.

This is noted, unfortunately the study did not collect data on the time a woman started using any method of the contraceptive.

Line 169: specify the confounders controlled for in the analysis

All the variables were considered important in explaining utilization of LAPM and were adjusted/controlled for in the regression model.

Line 166-173: Did the analysis control for the clustering effects? It is very important because of the survey design used.

We followed the design-based approach to the analysis of complex survey data and accounted for this while setting the data to the design and used “*svy*” commands in STATA.

We also “svy” set command in Stata while fitting the model to correct the standard errors.

Line 229-232: Authors argue that some supply-side factors are barriers to LAPM use, but I did not see that in the analysis result.

Thanks, we agree we do not have data on the supply-side factors, however, here we were giving a suggestions on how to increase uptake of the product. We have rephrased the statement

Thanks,

James Orwa
